# MO_x_/Rh Metallene with Energetic Interfaces as Efficient Bifunctional Electrocatalyst for Durable Water Splitting

**DOI:** 10.1002/advs.202511701

**Published:** 2026-03-30

**Authors:** Ruilong Wei, Yuner Lin, Shanshan Ye, Shuo Wang, Jing Gao, Chaochao Tao, Shengqian Wang, Pei Xiong, Hang Zhang, Hualan Xu, Shengliang Zhong

**Affiliations:** ^1^ Key Lab of Porous Functional Materials of Jiangxi Province College of Chemistry and Materials Jiangxi Normal University Nanchang China

**Keywords:** bifunctional electrocatalyst, metallene, overall water splitting

## Abstract

Developing excellent activity and stability bifunctional electrocatalysts for alkaline water splitting remains challenging. This work constructs ultrathin MO_x_/Rh metallene heterostructures (M = Fe, Co, Ni) via atmosphere‐controlled solvothermal synthesis, achieving uniform MO_x_ nanoclusters on Rh metallene with optimized interfaces. The FeO_x_/Rh catalyst demonstrates exceptional bifunctional activity, requiring only 1.53 V for overall water splitting at 10 mA cm^−2^ with robust durability. Leveraging the unique oxygen‐affinity of FeO_x_ and its strong electronic coupling with Rh, this study reveals the critical role of the high‐energy interface in accelerating the kinetics of the rate‐limiting four‐electron oxygen evolution reaction. Mechanistic insights reveal that FeO_x_ promotes water dissociation via optimized orbital hybridization, while electronically modulated Rh optimizes hydrogen adsorption (ΔG_H*_ = ‐0.057 eV) via d‐band downshifting. This interfacial engineering strategy enables efficient solar‐driven hydrogen production, offering a sophisticated paradigm for designing high‐performance bifunctional metallene catalysts.

## Graphical abstract

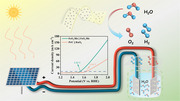

## 1 Introduction

Hydrogen energy has emerged as a cornerstone of future energy systems, offering exceptional energy density (142 MJ/kg) and carbon‐neutral combustion [1, 2]. Among various hydrogen production technologies, electrocatalytic water splitting stands out for its environmental compatibility and scalable potential [3, 4, 5]. However, the technology's commercialization faces fundamental challenges rooted in the dual half‐reaction mechanism: the hydrogen evolution reaction (HER) at the cathode and the oxygen evolution reaction (OER) at the anode [6, 7]. The kinetic limitations of the reactions, particularly the slow kinetics due to the four‐electron transfer mechanism of the OER, greatly reduce the overall efficiency of the water splitting devices [8, 9]. Moreover, in alkaline conditions, unlike acidic media where protons are supplied directly from the electrolyte, dissociation of water becomes a prerequisite for the HER [10, 11]. Current benchmarks rely on precious metal catalysts (i.e., Pt for HER, Ir/Ru oxides for OER) [12], yet their scarcity and prohibitive costs (> $30 000/kg for Ir) restrict practical implementation [13].

The rational design of catalyst surface kinetics can achieve local aggregation of active species and rapid desorption of products, enhancing surface kinetics and reaction kinetics [14]. Interface engineering has evolved into a powerful strategy for customising the performance of electrocatalysts by constructing heterostructures with optimized electronic coupling and synergistic functions. By precisely controlling the electronic structure and adsorption energy of the heterointerface [15], the HER/OER reaction pathways can be optimized simultaneously [16, 17]. During actual OER operation, although RuO_2_ exhibits higher activity than IrO_2_, it suffers from over‐oxidation of Ru sites to soluble RuO_4_^2−^ [18, 19]. An appropriate adsorption intensity optimizes reaction pathways and may theoretically balance activity with stability [20, 21]. The d‐band center of Rh is located between those of Ru and Ir, theoretically enabling a superior balance between activity and stability. Owing to its bifunctionality in both HER and OER, Rh metal serves as a single catalyst to simplify electrolyzer design. Moreover, rhodium‐based catalysts offer balanced adsorption energetics and corrosion resistance but suffer from severe aggregation under operational conditions [22, 23, 24]. For example, at the Rh‐Rh_2_O_3_ interface, metallic Rh promotes H adsorption while the oxide enhances OH binding, enabling an alkaline HER requiring only 63 mV to achieve 10 mA cm^−2^ [25]. Rhodium nanoparticles modified with phosphorus‐doped carbon nitride supports exhibit enhanced HER activity through interfacial electronic redistribution [26]. Rhodium nanoparticles supported on graphene oxide effectively reduce charge transfer resistance [27]. However, conventional Rh‐based catalysts suffer from nanoparticle aggregation (>5 nm) during cycling due to Ostwald ripening, leading to a >40% reduction in electrochemically active surface area [28]. This highlights the necessity for atomic dispersion and interface stabilization.

Recently, metallenes, a burgeoning class of 2D transition‐metal materials with atom‐thick thickness, have emerged as promising candidates for electrocatalysis due to their maximum atom utilization, unique electronic structures, and abundant coordinatively unsaturated surface sites [29, 30]. Among various 2D candidates, rhodium metallene has emerged as a premier platform for electrocatalysis due to its maximal atom utilization and unique electronic structure. Theoretically, Rh possesses a near‐zero hydrogen adsorption free energy (ΔG_H∗_), positioning it as a formidable rival to Pt for the hydrogen evolution reaction (HER) [31].

Despite these progresses, achieving a balance between hydrogen evolution and oxygen evolution on a single Rh‐based substrate remains a formidable task. Recently, it was reported that atomically dispersed MoO_x_ on Rh metallene could boost alkaline HER by accelerating the Volmer step [32]. However, MoO_x_ modification primarily addresses the hydrogen intermediates, leaving the more complex, four‐electron transfer OER process and the overall bifunctional synergy insufficiently explored. In this work, we diverge from previous monofunctional optimization strategies by coupling Rh metallene with 3d‐transition metal oxides [25, 33].

Here, we report a rational design of ultrathin MO_x_/Rh metallene (M = Fe, Co, Ni) heterostructures through a precise atmosphere‐controlled solvothermal synthesis. The selection of Rh metallene as a 2D substrate maximizes the exposure of active metallic sites, while the integration of oxyphilic 3d‐transition metal oxide nanoclusters is strategically engineered to bridge the kinetic gap of the OER and accelerate the HER. Unlike conventional physical mixtures or low‐density atomic doping, the established “high‐density energetic interfaces” facilitate profound interfacial charge redistribution and orbital hybridization. Specifically, the FeO_x_/Rh heterostructure leverages a dual‐site synergistic mechanism: the oxyphilic Fe species serve as efficient catalytic centers for oxygen intermediates during OER and promote water dissociation during HER, while the electronically modulated Rh surface optimizes the hydrogen adsorption/desorption energy by inducing a d‐band center downshift. This interfacial coupling not only prevents the over‐oxidation of Rh under high oxidative potentials but also significantly lowers the activation energy barriers for both half‐reactions. Consequently, the optimized FeO_x_/Rh catalyst achieves an exceptional bifunctional performance with a low cell voltage of 1.53 V at 10 mA cm^−2^ for overall water splitting, representing a significant advancement in transforming monofunctional noble‐metal metallenes into durable, high‐efficiency bifunctional electrocatalysts.

## 2 Results and Discussion

**FIGURE 1:**
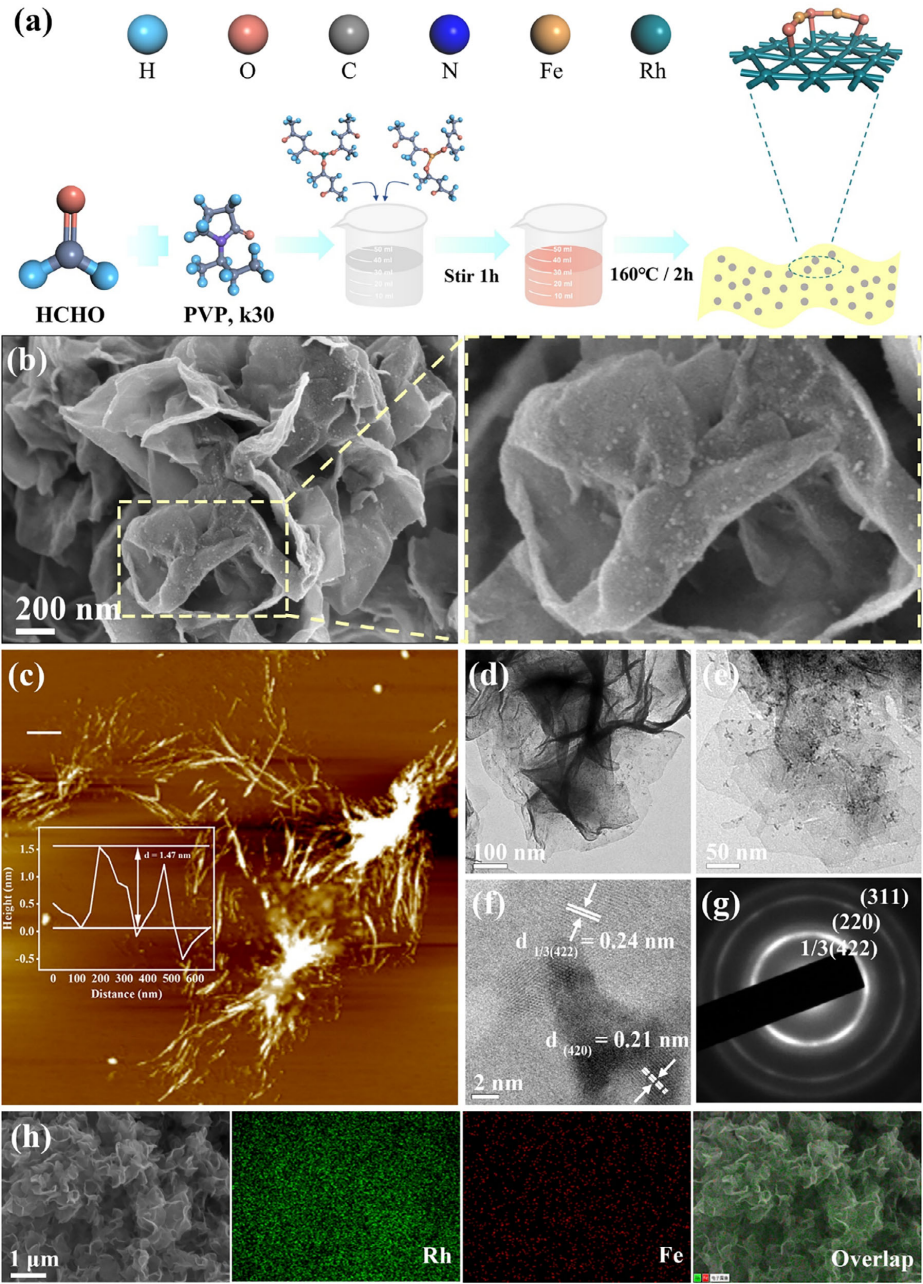
(a) Synthesis diagram of FeO_x_/Rh metallene (b) FESEM image of FeO_x_/Rh metallene (dashed box in inset shows local magnification). (c) AFM images of FeO_x_/Rh metallene. (d,e) Low magnification TEM images of FeO_x_/Rh metallene (f) HRTEM images of FeO_x_/Rh metallene. (g) SAED selected electron diffraction image of FeO_x_/Rh metallene. (h) Mapping elemental analysis image of FeO_x_/Rh metallene (Rh element in green, Fe element in red, and elemental overlay).

Based on the previous synthesis of Rh metallene [34]. The FeO_x_/Rh metallene was synthesized through a one‐pot solvothermal approach, as illustrated in Figure 1a. During the synthesis, Rh^3+^ ions were first reduced to Rh nanoparticles by formaldehyde. Subsequently, formaldehyde decomposition at elevated temperatures generated CO molecules, which were selectively adsorbed on Rh (111) surfaces. These adsorbed CO molecules acted as a confining agent to drive the 2D growth of Rh metallene [35]. The field emission scanning electron microscopy (FESEM) images (Figure 1b) reveal the unique nanoflower‐like morphology of the FeO_x_/Rh metallene, exhibiting micrometer‐scale lateral dimensions and exceptional mechanical flexibility, which are characteristic features of 2D nanomaterials. Higher magnification FESEM (Figure 1b inset) clearly shows the homogeneous distribution of 3–5 nm nanoparticles across the metallene surface, corresponding to FeO_x_ nanoclusters. The atomic force microscopy (AFM) analysis (Figure 1c) confirms the ultrathin nature of the material, with edge thickness measurements of merely 1.47 nm, consistent with previous reports on metallene structures [30, 36, 37]. The TEM investigations (Figure 1d,e) further confirm the 2D architecture, showing characteristic wrinkles and folds that demonstrate the material's mechanical flexibility. From the TEM image (Figure 1e), it can be seen that the surface of the nanosheet is uniformly distributed with small black dots of about 3–5 nm in diameter, which corresponds to the tiny particles observed in the FESEM image of (Figure 1b), which is possibly the iron oxide nanoclusters attached to its surface [38, 39, 40]. The high‐resolution transmission electron microscope (HRTEM) image in Figure 1f reveals distinct lattice fringes with spacings of 0.24 and 0.21 nm, corresponding to the Rh (1/3(422)) and Fe_2_O_3_ (420) crystallographic planes, respectively. The TEM characterization and XRD patterns of Rh metallene are shown in Figure S1 and S2. In the low‐magnification transmission electron microscope images, there are no corresponding nanocluster particles on the surface of Rh metallene. From the HRTEM images, both lattice fringes correspond to 1/3(422) of the Rh crystal plane. This provides direct evidence for the coexistence of metallic Rh and iron oxide phases at the atomic scale. Selected area electron diffraction (SAED) patterns (Figure 1g) exhibit concentric diffraction rings characteristic of face‐centered cubic (fcc) crystal structure, confirming the high crystallinity of the synthesized FeO_x_/Rh metallene. The EDS elemental mapping (Figure 1h) demonstrates the homogeneous spatial distribution of both Rh and Fe elements throughout the FeO_x_/Rh metallene architecture, with Rh serving as the primary metallic framework. Quantitative elemental analysis via ICP‐OES reveals precise stoichiometric ratios of 2.67 wt.% Fe and 78.7 wt.% Rh (Table S3), confirming the successful incorporation of iron oxide nanoclusters while maintaining rhodium as the dominant phase.

**FIGURE 2:**
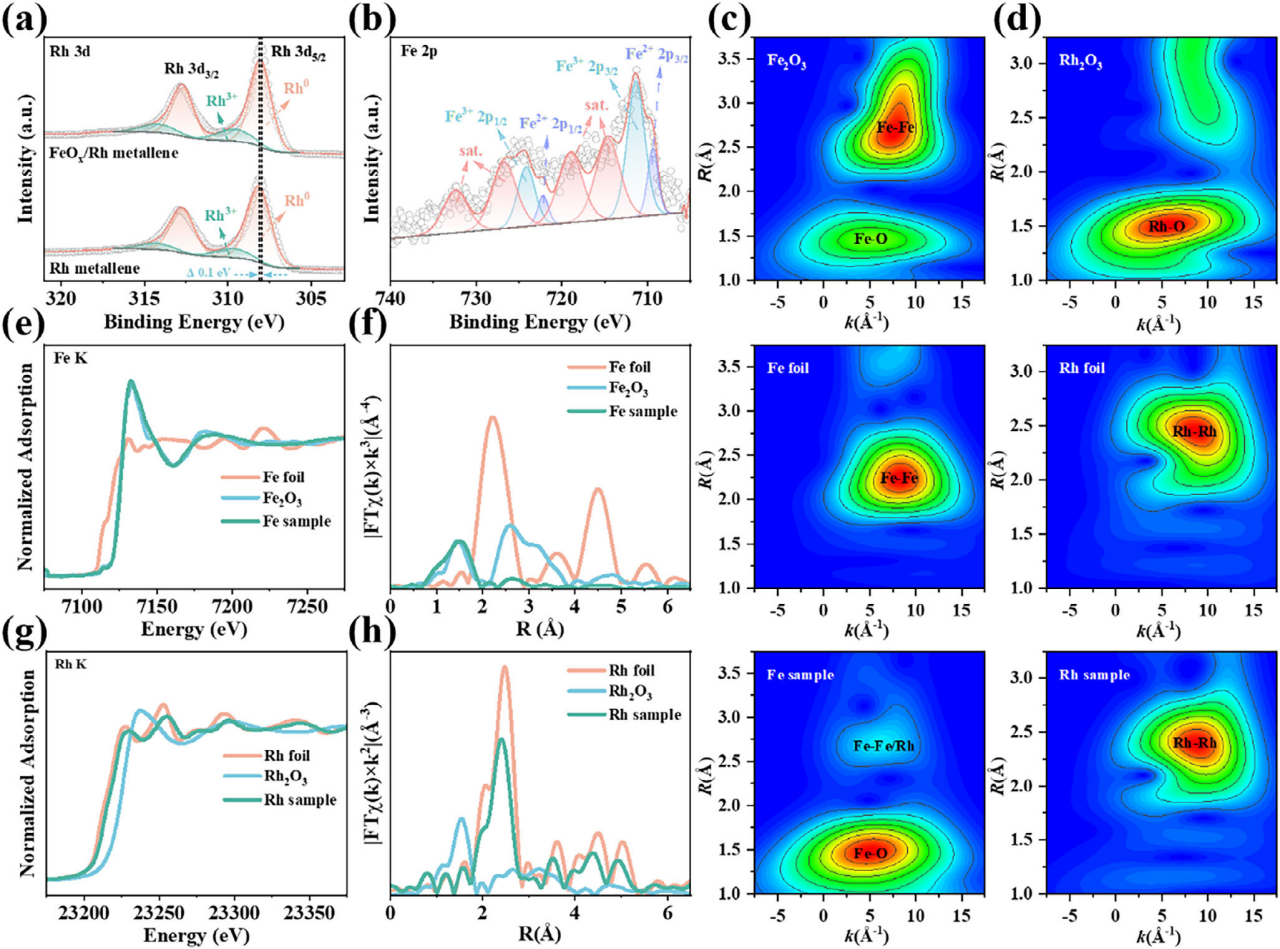
(a) Rh 3d XPS spectra of FeO_x_/Rh metallene and Rh metallene. (b) Fe 2p XPS spectra of FeO_x_/Rh metallene. (c) Wavelet transform of Fe k‐edge data Fe_2_O_3_, Fe foil, FeO_x_/Rh metallene (d) Wavelet transform of Rh k‐edge data Rh_2_O_3_, Rh foil, FeO_x_/Rh metallene. (e) Fe k‐edge XANES spectra. (f) EXAFS spectra of Fe foil, Fe_2_O_3_, and FeO_x_/Rh metallene. (g) Rh k‐edge XANES spectra. (f) EXAFS spectra of Rh foil, Rh_2_O_3_, and FeO_x_/Rh metallene.

Surface elemental composition and valence states of FeO_x_/Rh metallene were systematically investigated through XPS. The survey spectrum of FeO_x_/Rh metallene (Figure S3b) confirms the coexistence of Rh, Fe, and O elements, while pristine Rh metallene exhibits only Rh and O signals (Figure S6). High‐resolution Rh 3d spectra reveal distinct chemical states in both materials (Figure 2a). For FeO_x_/Rh metallene, the dominant doublet peaks at 308.0 eV (3d_5/2_) and 312.68 eV (3d_3/2_) correspond to metallic Rh(0), accompanied by minor oxidized components at 309.55 and 314.15 eV attributed to Rh^3+^ species. This indicates that on the surface of FeO_x_/Rh, metallene are mostly 0valent low valent species, accompanied by a small amount of oxidized to + 3 valent high valent species. In Rh metallene, the two high intensity peaks at 308.1 and 312.7 eV are assigned to Rh (0) 3d_5/2_ and Rh (0) 3d_3/2_, respectively, while the two weaker intensity peaks at 309.65 and 314.25 eV correspond to Rh (3+) 3d_5/2_ and Rh (3+) 3d_3/2_, respectively. Comparative analysis with pristine Rh metallene shows a notable 0.1 eV negative binding energy shift in the Rh 3d peaks of FeO_x_/Rh metallene, suggesting electron transfer to Rh species through interfacial interactions. The chemical state of iron was further elucidated through Fe 2p analysis (Figure 2b). The characteristic peaks at 711.31 and 724.11 eV correspond to Fe^3+^ 2p_3/2_ and Fe^3+^ 2p_1/2_. The characteristic peaks at 709.35 and 722.15 eV correspond to Fe^2+^ 2p_3/2_ and Fe^2+^ 2p_1/2_. The presence of a small amount of Fe^2+^ may be caused by the reducing atmosphere during the synthesis of metallene. Fine spectral analysis of Fe 2p confirmed the predominant existence of Fe^3+^. These spectral fingerprints jointly prove the successful formation of FeO_x_/Rh. The structural and electronic distinctiveness of the FeO_x_/Rh heterojunction establishes a fundamental departure from conventional 0D nanoparticle or bulk‐based catalysts (e.g., Pt/NiFe‐LDH [41]). Unlike recently reported W‐doped Rh metallene [42] or RhCu nanocrystals [43] that predominantly target hydrogen intermediate optimization, our 2D/0D architecture targets the bifunctional kinetic bottleneck. The atom‐thick nature of Rh metallene facilitates an exceptional degree of interfacial orbital hybridization, as evidenced by the strategic d‐band center downshift.

To probe the coordination structure of FeO_x_/Rh metallene, X‐ray absorption fine structure (XAFS) was systematically conducted. The normalized Fe K‐edge X‐ray absorption near edge structure (XANES) spectrum Figure 2e) reveals that the absorption edge and white line position of Fe in FeO_x_/Rh metallene align closely with those of Fe_2_O_3_, confirming a predominant Fe^3+^ valence state, consistent with XPS findings (Figure 2b) [44, 45]. The local coordination environment of the sample Fe atoms was further investigated by applying a Fourier transform to the Extended X‐ray Absorption Fine Structure (EXAFS) data through the k^3^ weighted χ(k) function, which transforms the data into R‐space. From the R‐space comparison and fitting results of Figure 2f (Figure S9 and Table S1), it can be observed that the first shell layer of Fe in the FeO_x_/Rh metallene is mostly Fe─O coordination at 1.5 Å, and the second shell layer is mostly Fe–Fe/Rh coordination at 2.6 Å. This could be the overlap of the second coordination shell layers of Fe‐Rh and Fe–Fe (Fe_2_O_3_, 2.97 Å), the small coordination number of 2.1 ± 1.0, suggesting that the FeO_x_ material is primarily dispersed on the surface of the Rh metallene. The K‐space fit data from Figure S9 show that the data fit well. Combined with wavelet transform (WT) data to further analyze the coordination environment of Fe [46], as shown in Figure 2c, the wavelet analysis of Fe element shows that the coordination environment of the sample is mainly Fe─O coordination in the first shell layer and Fe–Fe/Rh in the second shell layer.

For Rh coordination analysis, Rh K‐edge XANES spectra (Figure 2g) indicate that the absorption edge and white line intensity of FeO_x_/Rh metallene lie between metallic Rh foil and Rh_2_O_3_ but closer to Rh^0^, aligning with XPS observations of Rh mixed 0/+3 valence states [44, 45]. Further analysis of the EXAFS spectrum via Fourier transform (Figure 2h) shows that the samples contain mainly Rh–Rh ligands from the fitting results in R‐space, and the data are well fitted from K‐space (Figure S10 and Table S2). WT analysis of Rh elements (Figure 2d) reveals that Rh elements in FeO_x_/Rh metallene mainly contain Rh–Rh coordination environments [46]. Collectively, XAFS results demonstrate that FeO_x_/Rh metallene features a hybrid structure where Fe^3+^ oxide clusters are atomically dispersed on the Rh metallene surface through Fe‐O‐Rh linkages, and Rh retains its metallic lattice with minimal oxidation. This unique configuration facilitates electronic interactions between FeO_x_ and Rh, as evidenced by the XPS binding energy shifts, while preserving the catalytically active metallic framework of Rh.

**FIGURE 3:**
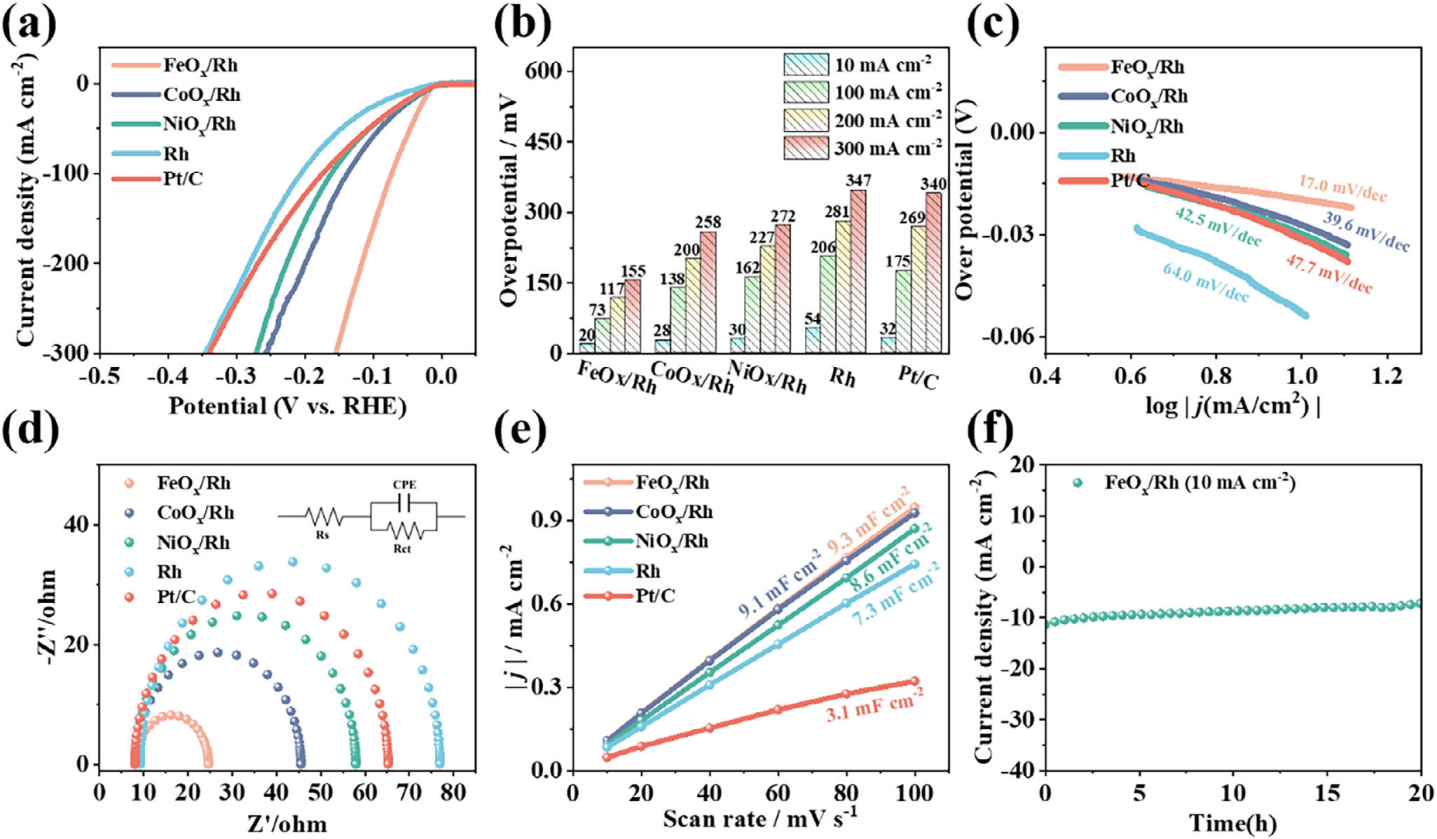
HER performance of the catalyst under 1 m KOH electrolyte (a) linear scanning voltammetry spectra (LSV). (b) Comparison of overpotentials at 10, 100, 200, and 300 mA cm^−2^ current density. (c) Tafel slope plots. (d) Electrochemical Impedance spectroscopy (EIS, inset shows equivalent circuit diagram). (e) Double Layer Capacitance (Cdl) plot. (f) Chronoamperometry plot.

The HER and OER catalytic activities of the samples were evaluated in a three‐electrode system using 1 m KOH electrolyte. All potentials were calibrated to the reversible hydrogen electrode (RHE) scale, with identical catalyst loadings on 3 mm diameter glassy carbon working electrodes. The HER activity of MO_x_/Rh under 1 m KOH was first explored to understand the effect of transition metals M (Fe, Co, Ni) on the catalytic performance of pure Rh metallene. Figure 3a shows the linear sweep voltammetry spectra of commercial Pt/C (20 wt.%) for FeO_x_/Rh, CoO_x_/Rh_,_ and NiO_x_/Rh, as well as pure Rh metallene and the benchmark catalyst (Pt/C). Among five catalysts, FeO_x_/Rh metallene possessed the highest HER activity, reaching current densities of 10 and 300 mA cm^−2^ at 20 and 155 mV, respectively, which was superior to CoO_x_/Rh (28 and 258 mV), NiO_x_/Rh (30 and 272 mV), Rh (54 and 347 mV), and commercial Pt/C (32 and 340 mV). The bar graph obtained from the LSV data (Figure 3b) shows the relationship between catalyst overpotential and current density. The Tafel slope of the catalysts was obtained by fitting the LSV curves to analyze the reaction kinetics of the catalysts in the HER process. As shown in Figure 3c the Tafel slope of FeO_x_/Rh is lower than that of CoO_x_/Rh (39.6 mV dec^−1^), NiO_x_/Rh (42.5 mV dec^−1^), Rh (64.0 mV dec^−1^), and Pt/C (47.7 mV dec^−1^), which is only 17.0 mV dec^−1^, suggesting that it follows the Volmer‐Tafel mechanism [47]. The loading of nanoparticles on the surface of metallene better modulates the electronic structure of the metallene surface [48], leading to its faster HER reaction kinetics. EIS analysis (Figure 3d) shows that the charge transfer resistance (R_ct_) of FeO_x_/Rh metallene is (16.52 Ω), which is lower than that of CoO_x_/Rh (37.39 Ω), NiO_x_/Rh (49.78 Ω), and Pt/C (57.18 Ω), indicating FeO_x_/Rh possesses a faster rate of electron transfer [49], which is favorable for its electrochemical catalytic performance. Accordingly, we measured the electrochemical active surface area (ECSA) of the catalysts using the double layer capacitance method (Figure 3e), and the measured C_dl_ were FeO_x_/Rh (9.3 mF cm^−2^), CoO_x_/Rh (9.1 mF cm^−2^), NiO_x_/Rh (8.6 mF cm^−2^), Rh (7.3 mF cm^−2^), and Pt/C (3.1 mF cm^−2^). The C_dl_ of FeO_x_/Rh metallene is the highest among several reference catalysts and shows a significant improvement relative to pure Rh metallene, suggesting that the uniform loading of nanoparticles on the surface of metallene facilitates the exposure of more active sites, which leads to better HER electrocatalytic performance. Moreover, the FeO_x_/Rh exhibits good electrochemical stability over a long period of time, as shown in Figure 3f, where the overpotential required to maintain the current density at 10 mA cm^−2^ changes only slightly. The hydrogen evolution performance of FeO_x_/Rh metallene in acidic and neutral media is shown in Figure S11.

**FIGURE 4:**
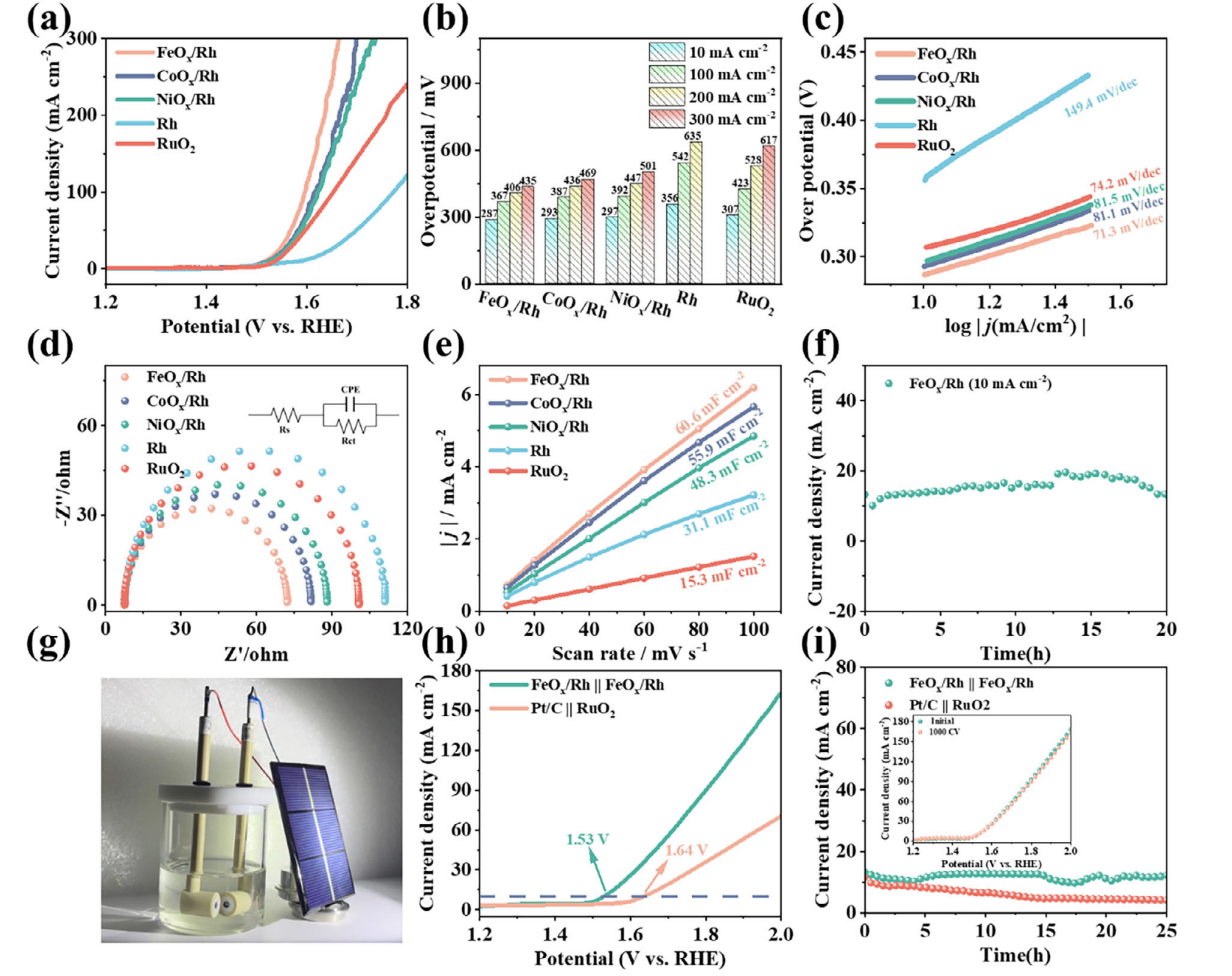
OER performance of catalysts under 1 m KOH electrolyte (a) linear sweep voltammetry spectra. (b) Comparison of overpotentials at 10, 100, 200, and 300 mA cm^−2^ current density. (c) Tafel slope plot. (d) EIS plot (inset shows equivalent circuit diagram). (e) C_dl_ plot. (f) Chronoamperometry plot. (g)Simplified diagram of a solar‐assisted water electrolysis unit. (h) Overall water splitting curve. (i) Overall water splitting chronoamperometric curves (inset shows the accelerated durability test (ADT) plot).

Due to the excellent HER performance of the material, we also tested the performance of FeO_x_/Rh metallene in oxygen evolution reaction (OER) under 1 m KOH electrolyte. As shown in Figure 4a, FeO_x_/Rh metallene achieved a current density of 10 mA cm^−2^ at 287 mV in a LSV test, which was superior to CoO_x_/Rh metallene (293 mV), NiO_x_/Rh metallene (306 mV), Rh (321 mV), and commercial RuO_2_ (305 mV) catalysts. Notably, FeO_x_/Rh metallene only requires an overpotential of 367 mV to reach a current density of 100 mA cm^−2^ (Figure 4b), while pure Rh metallene has a current density of only 21.5 mA cm^−2^ at this overpotential, indicating that nanoparticle loading on the surface of metallene can greatly improve its OER performance. Further analyzing the Tafel slopes of the catalysat in this point, as shown in Figure 4c, FeO_x_/Rh metallene possesses the lowest Tafel slope (71.3 mV dec^−1^), which is much lower than that of Rh metallene (157.9 mV dec^−1^), and even lower than that of the baseline catalyst, RuO_2_ (73.0 mV dec^−1^), which also indicates that FeO_x_/Rh metallene possesses faster kinetics. The EIS impedance spectra (Figure 4d) also show that FeO_x_/Rh metallene possesses a faster electron transfer rate compared to CoO_x_/Rh metallene, NiO_x_/Rh metallene, and Rh metallene, allowing for faster electron transfer during the reaction. The ECSA of the catalysts was further evaluated using the double‐layer capacitance method. The measured C_dl_ values (Figure 4e) were FeO_x_/Rh metallene (60.6 mF cm^−2^), which was much larger than the value of Rh metallene (31.1 mF cm^−2^), and RuO_2_ (15.3 mF cm^−2^), respectively, suggesting that they were able to expose more active sites, which could help to improve the OER electrocatalytic ability of FeO_x_/Rh metallene in 1 m KOH alkaline electrolyte. A Chronoamperometry plot of FeO_x_/Rh metallene during OER reaction is shown in Figure 4f. As shown in the figure, the reaction current density is maintained above 10 mA cm^−2^. The electrocatalytic performance of FeO_x_/Rh, CoO_x_/Rh, and NiO_x_/Rh metallene with different Fe(acac)_3_, Co(acac)_3_, and Ni(acac)_2_ additions are shown in Figures S13–S24.

Capitalizing on the dual HER/OER excellence of FeO_x_/Rh metallene, a two‐electrode alkaline electrolyzer was constructed by depositing the catalyst onto 5 mm × 5 mm carbon fiber paper (CFP) for both cathode and anode. The air‐dried electrodes were assembled into a full cell configuration to evaluate their bifunctional activity in 1 m KOH. As illustrated in Figure 4g, a small commercial solar panel with an operating voltage of 1.5 V is able to drive the electrolyzer for overall water splitting, and the evolution of hydrogen and oxygen can be clearly observed (as shown in Figure S25 and Videos S1 and S2). This solar‐assisted overall water splitting device has the potential to be applied to distributed energy storage technologies [50]. Remarkably, the FeO_x_/Rh‐based electrolyzer requires only 1.53 V to achieve 10 mA cm^−2^ (Figure 4h), substantially outperforming the benchmark Pt/C || RuO_2_ system (1.64 V). Such low operational voltage not only enhances energy efficiency but also supports compact, sustainable device designs. The stability of the electrolyzer was tested by ADT and Chronoamperometry, as shown in the inset of Figure 4i. There was only a slight decrease in the activity of the overall water splitting electrolyzer after 1000 cycles of CV test. The long‐term stability of the two‐electrode electrolyzer was tested by the chronoamperometry method, in which the current density was more stable and always maintained above 10 mA cm^−2^ over a long period of 25 h. Chronopotentiometry was employed to further demonstrate the stability of FeO_x_/Rh metallene, as shown in Figure S26. The characterization after the stability test shows that the stability of FeO_x_/Rh metallenes is well, as shown in Figures S27–S31. In contrast, the Pt/C || RuO_2_ electrolyzer, as a comparison, showed a continuous decrease in the activity from the beginning of the reaction, and was not able to maintain a current density of 10 mA cm^−2^. The FeO_x_/Rh/CFP || FeO_x_/Rh/CFP electrolyzer synergizes exceptional efficiency (1.53 V @ 10 mA cm^−2^) with unparalleled durability, positioning it as a transformative candidate for scalable green hydrogen production.

**FIGURE 5:**
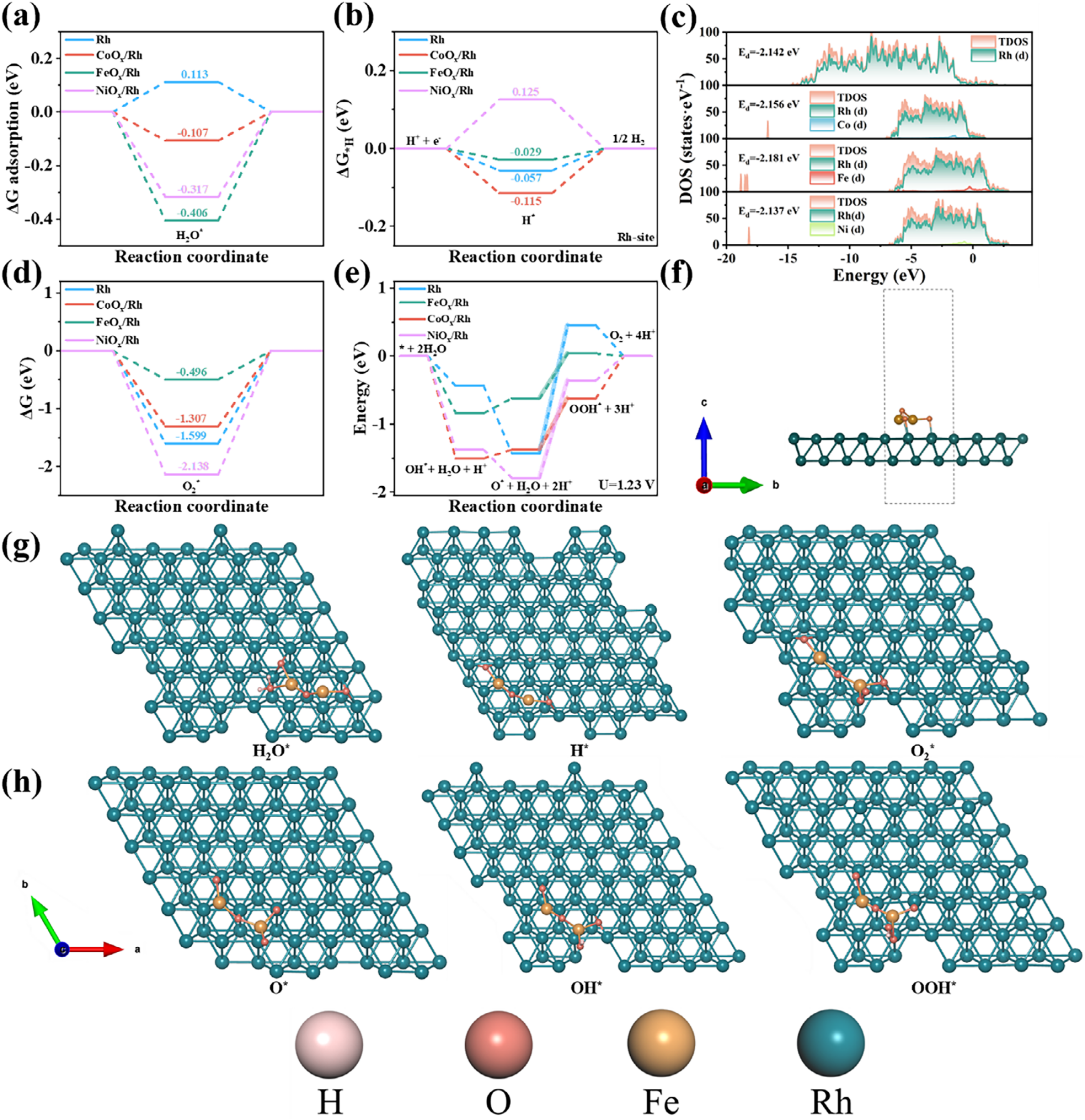
(a) Gibbs free energy of dissociation for water. (b) Calculated Gibbs free energy plot for HER at equilibrium potential. (c) Projected density of states. (d) Calculation of Gibbs free energy binding energy for adsorbed O_2_. (e) Calculated free energy step diagram relative to RHE at potential U = 1.23 V (The bolded dashed line in the figure is the potential determining step (PDS). (f) Structural optimized configurations of FeO_x_/Rh metallene. (f) Structural optimized configurations of FeO_x_/Rh metallene. (g) Configurations of H_2_O^*^、H^*^、and O_2_^*^ intermediates adsorbed on FeO_x_/Rh metallene surface (H^*^ intermediate adsorbs on Rh site). (h) Configurations of OER intermediates adsorbed on FeO_x_/Rh metallene surface.

To elucidate the origin of the superior HER/OER bifunctionality in FeO_x_/Rh metallene, DFT calculations were conducted on four structural models: Rh metallene, FeO_x_/Rh, CoO_x_/Rh, and NiO_x_/Rh. In alkaline HER, H_2_O has to be adsorbed to the active site first [51], hence, the adsorption energy of H_2_O on the catalyst surface was investigated. As shown in Figure 5a, the calculated adsorption energy of H_2_O on FeO_x_/Rh is ‐0.406 eV, which is larger than that on CoO_x_/Rh (‐0.107 eV), NiO_x_/Rh (‐0.317 eV), and Rh (0.113 eV), which suggests that there is a stronger adsorption of H_2_O on FeO_x_/Rh, which can promote the Volmer steps.

Subsequent hydrogen adsorption Gibbs free energy, as shown in Figure 5b, FeO_x_/Rh metallene is closer to the thermal neutral hydrogen adsorption free energy than the other two metallenes, which explains why FeO_x_/Rh metallene possesses the best activity in the HER process. Rh, as a sort of noble metal, has a high d band center position close to the Fermi energy level, which leads to its overly strong adsorption with hydrogen intermediates, which tends to result in the difficulty of desorption of H^*^ [52, 53], and the generated hydrogen cannot be detached from the surface of the catalyst on time. PDOS diagrams of FeO_x_/Rh, CoO_x_/Rh, NiO_x_/Rh, and Rh metallene were calculated and mapped. As shown in Figure 5c. The d band centers of FeO_x_/Rh metallene were shifted downward compared to Rh metallene, which suggests that FeO_x_/Rh metallene are more suitable for the adsorption of H^*^ intermediates during the reaction process and that the generated H_2_ can be detached from the surface of Rh quickly, thus accelerating the reaction rate. During the OER process, if the adsorption of O_2_ with the catalyst surface is too strong, which will lead to O_2_ occupying the active sites, causing a decrease in activity if it cannot be detached from the catalyst surface in time after O_2_ generation, the Gibbs free energy of oxygen molecules adsorbed on the catalyst surface was studied. As shown in Figure 5d, the Gibbs free energy of FeO_x_/Rh metallene (‐0.496 eV), which is lower than that of CoO_x_/Rh metallene (‐1.307 eV), NiO_x_/Rh (‐2.138 eV), and Rh metallene (‐1.599 eV), suggesting generated oxygen molecules are more readily desorbed from the surface of FeO_x_/Rh, thus allowing the active site to regenerate quickly. The four‐electron transfer mechanism proposed by Nørskov et al., was used to investigate the relationship between various reactive intermediates and the catalytic surface during alkaline OER (oxygenated intermediates OH^*^, O^*^, and OOH^*^ adsorbed on transition metal sites). As shown in Figure 5e, the RDS of all four studied models is the step of O^*^ to generate OOH^*^, in which the RDS value of FeO_x_/Rh metallene is only 0.66 eV, which is much lower than that of Rh metallene of 1.88 eV, while the RDS values of the remaining two CoO_x_/Rh and NiO_x_/Rh metallene are 0.75 and 1.44 eV, indicating that in the procession of OER reaction, FeO_x_/Rh metallene alkenes possess the lowest energy barrier, which favors the reaction. As shown in Figure S32. for Rh metallene, the step of conversion of O^*^ to OOH^*^ shows a free energy change of 3.113 eV, indicating the slow kinetics of OER procession. In contrast, the adsorption free energies of FeO_x_/Rh, CoO_x_/Rh, and NiO_x_/Rh metallene for oxygenated intermediates in this step were reduced to 1.891, 1.982, and 2.665 eV, respectively, which match with the experimental results, further illustrating that the transition metal oxide nanoclusters loading on the surface of Rh metallene could improve the catalytic activity. Figure 5f shows the structural optimization model of FeO_x_/Rh metallene [54], Figure 5g shows the adsorption structure model of FeO_x_/Rh metallene for adsorption of H_2_O^*^, H^*^, and O_2_^*^ intermediates, and Figure 5h shows the adsorption structure model of FeO_x_/Rh metallene for adsorption of OER intermediates. Overall, FeO_x_ nanoclusters can form a synergistic interaction with Rh, which facilitates the adsorption of H_2_O on the catalyst surface, the adsorption of H^*^, and the desorption of O_2_, and optimizes the energy required for the reaction, leading to the facilitation of the reaction, which explains the excellent alkaline HER, as well as OER, properties of FeO_x_/Rh metallene observed.

As shown in Figure S36, the ex situ Raman spectra of FeO_x_, Rh, and FeO_x_/Rh exhibit distinct differences. The characteristic bands of FeO_x_ at 217, 286, 400, 492, and 614 cm^−1^ correspond to the typical lattice vibrations of iron oxides (A^1^_g_/E_g_ phonon modes), consistent with α‐Fe_2_O_3_ [55]. Owing to the nanocluster morphology of FeO_x_, these phonon modes exhibit noticeable peak shifts and broadening relative to bulk α‐Fe_2_O_3_, which can be attributed to nanoscale phonon confinement as well as lattice defects and strain effects [56, 57]. Rh shows only a weak Raman feature at ∼264 cm^−1^ due to its intrinsically low Raman scattering efficiency.

To further substantiate the origin of the vibrational features in FeO_x_/Rh, a Lorentzian deconvolution was performed on the broad Raman band centered at ∼567 cm^−1^ (Figure S37). The deconvolution reveals that this feature is a convolution of multiple modes: the peaks at 216, 281, 398, 499, and 608 cm^−1^ are characteristic of the A^1^_g_ and E_g_ symmetries of α‐Fe_2_O_3_ (hematite) [58]. Most significantly, the distinct fitted peak at 551 cm^−1^ is assigned to the E_g_ mode of nanocrystalline Fe_3_O_4_ (magnetite) [59]. The emergence of this mixed‐valence phase at the interface is independently corroborated by the XPS analysis, which identifies a coexistence of Fe^2+^ and Fe^3+^ species. This interfacial structural evolution, induced by the strong electronic coupling with the Rh metallene substrate, lowers the coordination symmetry of Fe─O bonds and creates highly active bridge‐oxygen sites for water dissociation [60].

The potential‐dependent in situ OER Raman spectra further reveal distinct activation behaviors. For pure FeO_x_ (Figure S38), the Fe─O vibration at ∼612 cm^−1^ begins to blueshift only at potentials above ∼1.72 V, indicating that its Fe─O coordination environment undergoes appreciable modification only at relatively high anodic bias. In contrast, the ∼567 cm^−1^ vibration of FeO_x_/Rh starts to blueshift as early as ∼1.12 V (Figure S39), and exhibits a much stronger potential dependence compared to pristine FeO_x_, suggesting that Fe sites in the composite experience potential‐induced structural/electronic reorganization at substantially lower potentials. The Raman spectrum of Rh shows negligible changes under OER conditions (Figure S40). Overall, the in situ spectra demonstrate a stronger potential dependence and earlier activation of Fe─O‐related vibrations in FeO_x_/Rh compared to FeO_x_ [61].

These observations collectively indicate that Rh strongly modulates the local structure and electronic configuration of FeO_x_. The suppression of FeO_x_ lattice phonons and the appearance of the 567 cm^−1^ band in FeO_x_/Rh point to pronounced interfacial coupling between FeO_x_ and Rh, which alters Fe─O bond strength, symmetry, and local coordination, an effect widely reported in transition‐metal‐oxide/metal heterointerfaces. FeO_x_/Rh also exhibits an earlier onset of Fe─O vibrational evolution and a significantly stronger change in spectral characteristics compared with FeO_x_, implying that Rh facilitates charge redistribution at Fe sites and lowers the energetic barrier for potential‐induced structural activation. Consequently, Rh accelerates the electrochemical structural evolution of Fe sites, enabling their transition into OER‐competent coordination environments at significantly lower potentials. Consequently, Rh accelerates the electrochemical structural evolution of Fe sites, enabling their transition into OER‐competent coordination environments at significantly lower potentials [62, 63].

## 3 Conclusion

In summary, we have strategically engineered a bifunctional FeO_x_/Rh metallene electrocatalyst by anchoring FeO_x_ nanoclusters onto atomic‐layer Rh sheets via an atmosphere‐controlled solvothermal route. This high‐energy interface overcomes the intrinsic kinetic limitations of pure Rh in alkaline media, delivering exceptional HER overpotentials (20 mV at 10 mA cm^−2^) and significantly enhanced OER activity with a minimal Tafel slope (71.3 mV dec^−1^). When integrated into a two‐electrode electrolyzer, the FeO_x_/Rh system requires only 1.53 V to drive 10 mA cm^−2^, markedly surpassing the commercial Pt/C || RuO_2_​ benchmark (1.64 V) and outperforming previously reported Rh‐based monofunctional catalysts. Computational and spectroscopic insights reveal that the FeO_x_ nanoclusters and Rh metallene synergistically modulate the interfacial electronic structure through profound orbital hybridization. This effect not only optimizes water dissociation and H^∗^ binding (ΔG_H∗_≈0 eV) for HER but also bridges the kinetic gap for four‐electron OER processes by preventing Rh over‐oxidation and lowering intermediate adsorption energies. The successful solar‐assisted hydrogen production further underscores the practical viability of this interface‐engineering strategy. Our work demonstrates that rational interfacial coupling between 2D noble‐metal metallenes and 0D transition‐metal oxides provides a universal and efficient pathway for developing robust bifunctional catalysts for sustainable green hydrogen production.

## Conflicts of Interest

The authors declare no conflicts of interest.

## Supporting information




**Supporting File 1**: advs74950‐sup‐0001‐SuppMat.docx.

**Supporting File 2**: advs74950‐sup‐0002‐VideoS1.mp4.

**Supporting File 3**: advs74950‐sup‐0003‐VideoS2.mp4.

## Data Availability

The data that support the findings of this study are available from the corresponding author upon reasonable request.
