# Supplementary material for: MOx/Rh Metallene with Energetic Interfaces as Efficient Bifunctional Electrocatalyst for Durable Water Splitting
Source: Adv Sci (Weinh). 2026 Mar 30;13(32):e11701. doi: 10.1002/advs.202511701 (PMC13252626; doi:10.1002/advs.202511701)
Supplement: Supplementary file 1 — Supporting File 1: advs74950‐sup‐0001‐SuppMat.docx. [file ADVS-13-e11701-s003.docx]

Supporting Information

MO_x_/Rh metallene with energetic interfaces as efficient bifunctional electrocatalyst for durable water splitting

Ruilong Wei, Yuner Lin, Shanshan Ye, Shuo Wang, Jing Gao, Chaochao Tao, Shengqian Wang, Pei Xiong, Hang Zhang*, Hualan Xu*, Shengliang Zhong*

Dedication

R. Wei, Y.Lin, S. Ye, S. Wang, J. Gao, C. Tao, S. Wang, P. Xiong, H. Zhang, H. Xu, S. Zhong

Institution:Key Lab of Porous Functional Materials of Jiangxi Province, College of Chemistry and Materials.

Address:Jiangxi Normal University, Nanchang 330022, China.

E-mail: [slzhong@jxnu.edu.cn](mailto:slzhong@jxnu.edu.cn)

# Experimental section

## Materials

Rhodium(III) acetylacetonate (Rh(acac)_3_, 98%, Beijing Innochem Technology Co., Ltd.); Iron(III) acetylacetonate (Fe(acac)_3_, 98%, Beijing Innochem Technology Co., Ltd.); Cobalt(III) acetylacetonate (Co(acac)_3_, 98%, Beijing Innochem Technology Co., Ltd.); Nickel(II) acetylacetonate (Ni(acac)_2_, 96%, Beijing Innochem Technology Co., Ltd.); Polyvinylpyrrolidone (PVP, K30, average molecular weight: 40,000, Beijing Innochem Science and Technology Co., Ltd.); Formaldehyde solution (HCHO, 37%, Teichai (Shanghai) Kasei Industry Development Co., Ltd.); Nafion perfluorinated ion-exchange resin (5 wt%, Thermo Fisher Scientific).

## Synthesis

Based on the previous synthesis of Rh metallene, we improved the synthesis method to synthesize FeO_x_/Rh metallene. First, 24 mg of Rh(acac)_3_ and 3 mg of Fe(acac)_3_ were dispersed in 12 mL of HCHO solution and stirred for 10 min until fully dissolved to form Solution A. 120 mg of PVP was dispersed in 6 mL of HCHO solution, stirred for 10 min, and dissolved completely to form Solution B. Solutions A and B were then combined as Solution C, followed by vigorous stirring for 1 h. The mixture was transferred to a PTFE lined autoclave and heated at 160°C for 2 h. After cooling to room temperature, the product was washed three times with ethanol and dried under vacuum at 60°C overnight to obtain FeO_x_/Rh metallene**.** For comparison, Rh metallene was synthesized under identical conditions without adding Fe(acac)_3_. FeO_x_ was synthesized under identical conditions without adding Rh(acac)_3_.

Similarly, CoO_x_/Rh and NiO_x_/Rh metallene were prepared by replacing Fe(acac)_3_ with Co(acac)_3_ and Ni(acac)_2_, respectively. To study compositional effects, FeO_x_/Rh, CoO_x_/Rh, and NiO_x_/Rh metallene with varying metal ratios (4:1, 6:1, 8:1, 10:1, 12:1) were synthesized by adjusting the precursor quantities(Fe(acac)_3_: 6 mg, 4 mg, 3 mg, 2.4 mg, 2 mg, Co(acac)_3_, and Ni(acac)_2_ analogously)

## Selective site poisoning experiment

Specific molecules are used to selectively poison FeO_x_ or Rh sites, and loss-of-function experiments are performed to disprove the role of different sites in the catalytic cycle. After the HER test, a low concentration of a poisoning agent (1 mM KSCN or Na_3_PO_4_) is added to the 1 M KOH electrolyte. After the current stabilizes, LSV tests are performed again to observe changes in HER performance.

## Characterization

The products were characterized by X-ray diffraction (XRD) using a Rigaku Ultima IV X-ray diffractometer with Cu-Kα radiation (λ = 1.54178 Å) over a scanning range of 5° to 80° (2°/min) to determine their crystalline phase. Sample thickness was analyzed using a Bruker Dimension Edge (USA). The morphology was examined using Hitachi S3400N and Zeiss Sigma 300 scanning electron microscopes (SEM), equipped with energy dispersive X-ray spectroscopy (EDS). The microscopic morphology, crystal spacing, and related features were characterized by transmission electron microscopy (TEM, JEOL 2100). Surface elemental composition and chemical states were analyzed via X-ray photoelectron spectroscopy (XPS, Shimadzu AXIS SUPRA), and bulk elemental content was quantified using inductively coupled plasma optical emission spectrometry (ICP-OES, Agilent 5110). X-ray absorption fine structure spectroscopy (XAFS) was performed at the BL14B2 beamline of Spring8. Electrochemical performance was evaluated using a CHI760E electrochemical workstation (Shanghai Chenhua). Raman spectroscopic analysis was carried out using a Thermo Fisher Scientific instrument with a 532 nm excitation source, while in situ Raman measurements were conducted using a Renishaw micro Raman spectrometer, featuring a 532 nm laser excitation within a specialized single-chamber electrochemical operando Raman cell designed by In situ High-tech.

## Electrochemical measurements

For electrochemical testing, Electrochemical inks were prepared by mixing 5 mg catalyst with 960 μL ethanol and 40 μL 5 wt% Nafion in a 2 mL centrifuge tube, followed by 40 min sonication with manual shaking at 5 min intervals to achieve uniform dispersion For working electrode preparation, a 3 mm diameter L-type glassy carbon electrode (0.070686 mm²) was polished with alumina polishing powder, flushed clean and fully dried, then dropped with 5 μL electrochemical ink (5 μL in 2 times), allowing complete drying before electrochemical testing. Durability test electrodes were prepared by loading 200 μL of the ink onto 1 cm² hydrophilic carbon fiber paper (CFP) followed by room temperature drying. All electrochemical tests used 1 M KOH electrolyte with a three-electrode system consisting of glassy carbon or CFP as the working electrode, graphite as the counter electrode, and a saturated calomel electrode (Hg/HgO) as the reference electrode. Electrode potentials were converted to reversible hydrogen electrodes (RHE) using E(RHE)=E(SCE)+0.0591pH+0.24, with polarization curves receiving 90% IR compensation.

In a 0.5 M H₂SO₄ solution saturated with Ar, cyclic voltammetry (CV) curves for hydrogen underpotential deposition (H_upd_) were obtained using a potential window of -0.175 V to 1.024 V vs. RHE and a scan rate of 100 mV s⁻¹. The electrochemical active surface area (ECSA) was calculated by dividing the area of the H_upd_ peak (0.081–0.473 V vs. RHE) by the total mass of rhodium.

$$ECSA=\frac{S_{H}/V}{0.21(mC*{cm}^{-2})*M_{Rh}}$$

In the formula, S_H_ represents the peak area, V represents the sweep rate, with units of V/s, 0.21 represents the coefficient, with units of mC*cm^-2^, M_Rh_ represents the mass of the electrode surface Rh, with units of g.

## DFT calculations

All theoretical calculations are based on Density Functional Theory (DFT+U) and are performed through the Vienna Ab-initio Simulation Package (VASP). Structural modeling was performed by Materials Studio (MS) to create an expanded 3 × 3 × 1 Rh (111) faceted supercell with a vacuum layer of 15 Å thickness in the z-direction in order to minimize the interactions between adjacent lattices. The exchange correlation function is handled using the generalized gradient approximation (GGA-PBE) method. The cut off energy for the relaxation process was set to 450 eV. Calculations stopped when the energy and force were less than 10-4 and -0.05 eV/Å, respectively. A 4 × 4 × 1 grid of K-points centered on the Gamma point was set. In this study, the U values for Rh, Fe, Co, and Ni were set to 4 eV.

To determine the rate determining step and relative OER activities of FeO_x_/Rh, CoO_x_/Rh, NiO_x_/Rh, and Rh, the free energies of adsorption of the three intermediates (O^*^, OH^*^, and OOH^*^) were calculated, and the adsorption energies were calculated using the following equations:

$E_{ads}=E_{total}-\left( E_{surface}+E_{adsorbate} \right)$ (1)

Express:

$\Delta E_{ads}\left( O^{*} \right)=E\left( *O \right)-E\left( * \right)-\left( EH_{2}O-EH_{2} \right)$ (2)

$\Delta E\left( {OH}^{*} \right)=E\left( *OH \right)-E\left( * \right)-\left( EH_{2}O-\frac{1}{2}EH_{2} \right)$ (3)

$\Delta E\left( {OOH}^{*} \right)=E\left( *OOH \right)-E\left( * \right)-\left( 2EH_{2}O-\frac{3}{2}EH_{2} \right)$ (4)

Where E(∗), E(O^*^), (OH^*^), and E (OOH^*^) are the surface energy and the total surface energy of adsorption by the intermediates of O^*^, OH^*^, and OOH^*^, respectively, and $EH_{2}$,$EH_{2}O$ are the energies of the molecules in the gaseous state (from thermodynamic database).

Based on the above calculated adsorption energies, the free energy of adsorption was calculated as follows:

$\Delta G_{ads}=\Delta E_{ads}+\Delta ZPE-T\Delta S$ (5)

Where ΔE_ads_ is the energy change calculated above, and ΔZPE, T, and ΔS are the zero point energy, temperature (298.15 K), and entropy change values, respectively. Zero point energies and entropies for the OER intermediates were calculated from vibrational frequencies (the substrates were fixed during the calculations and adsorption vibrational modes were allowed to be calculated). ZPE and entropy for gas phase molecules were obtained from the thermodynamic database.


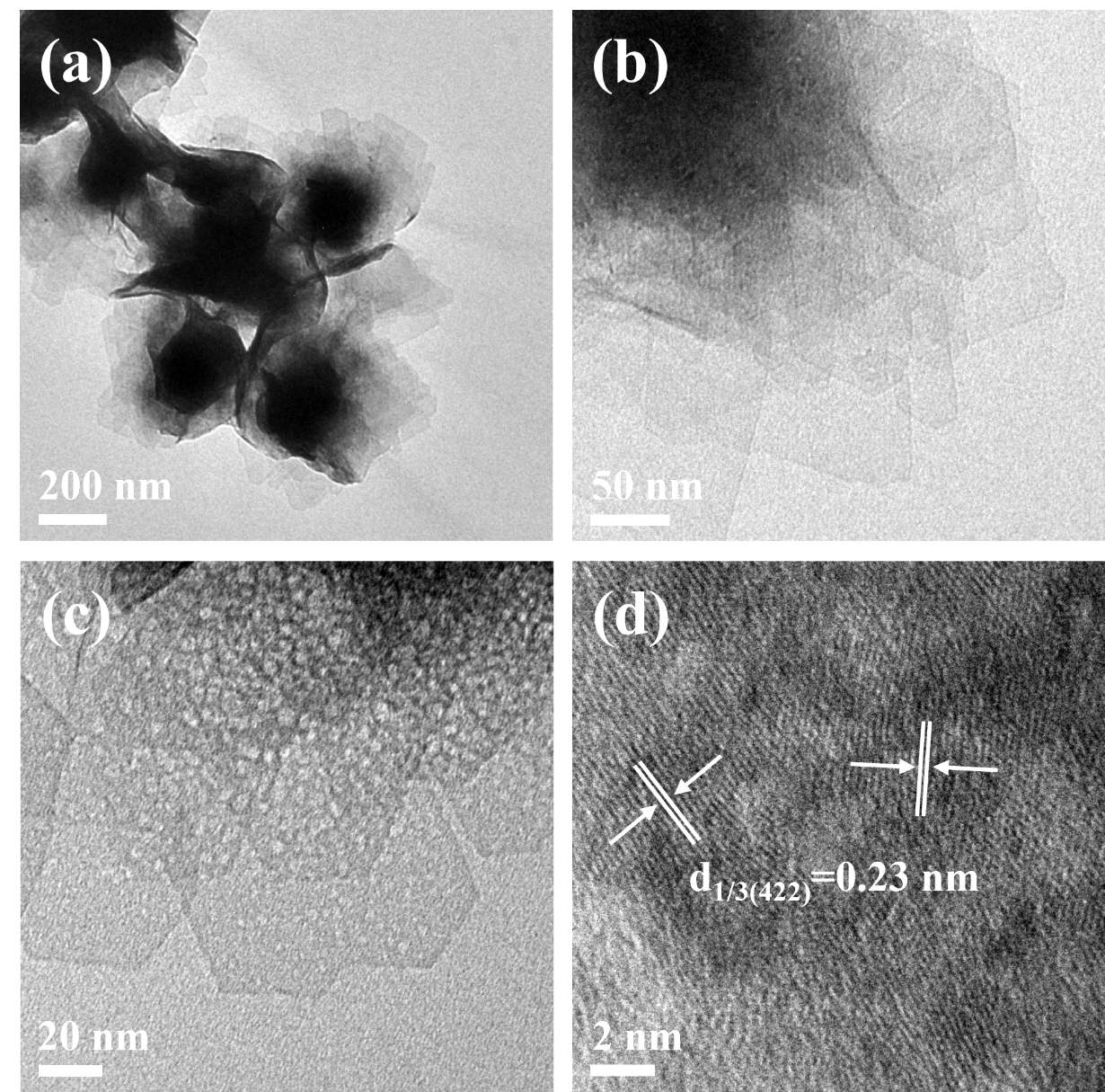


**Figure S1.** (a-b) Low magnification TEM images of Rh metallene (c-d) HRTEM images of Rh metallene.


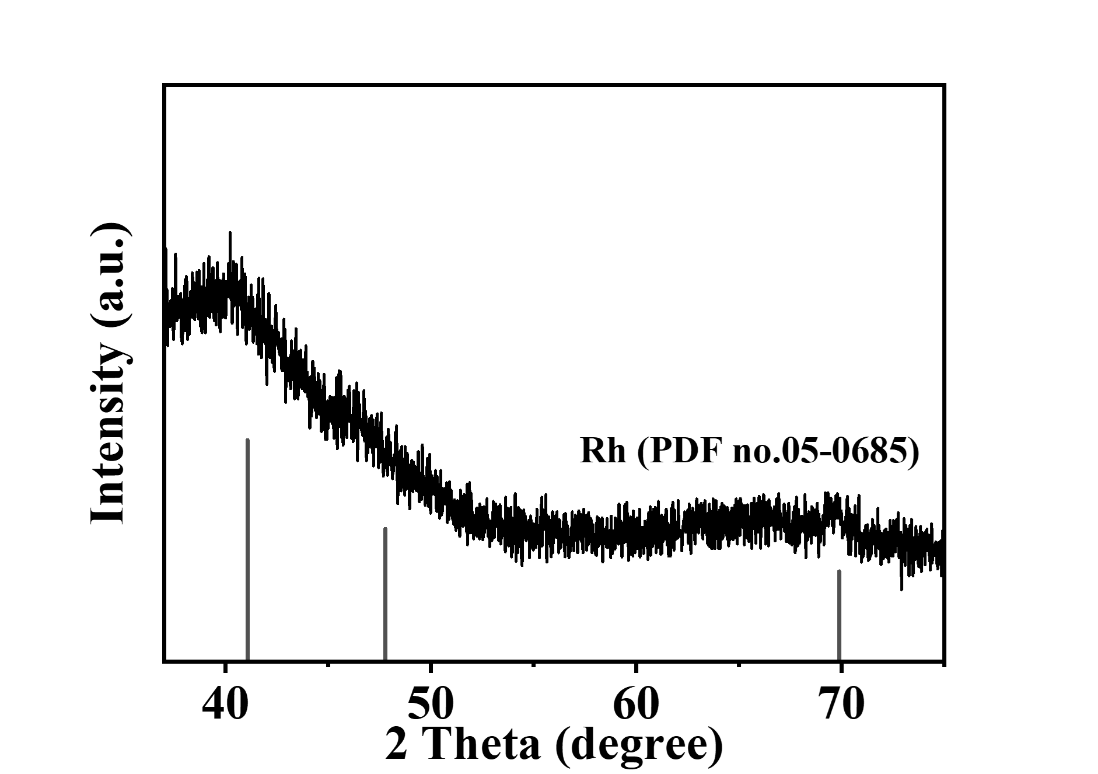


**Figure S2.** XRD pattern of Rh metallene.


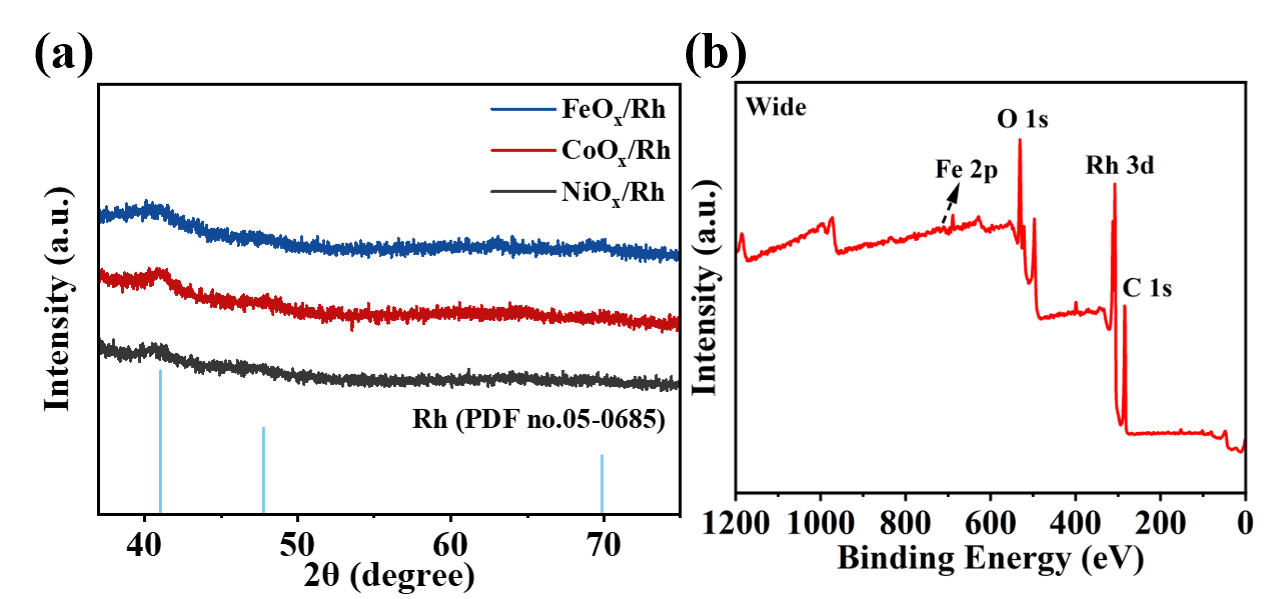


**Figure S3.** (a) XRD pattern of MO_x_/Rh (M=Fe, Co, Ni). (b) Survey XPS spectra of FeO_x_/Rh metallene.


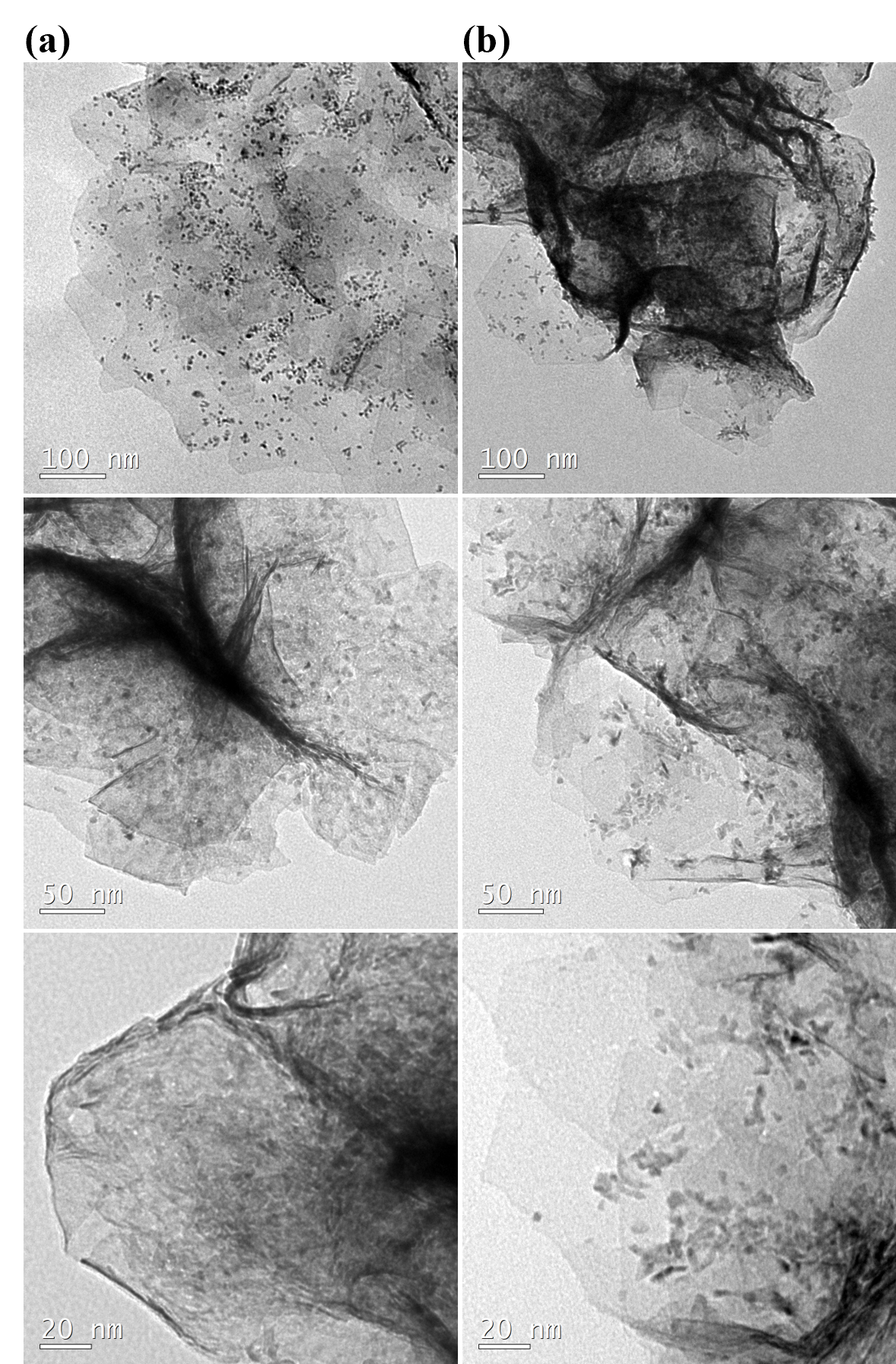


**Figure S4.** (a) Low magnification TEM images of CoO_x_/Rh metallene. (b) Low magnification TEM images of NiO_x_/Rh metallene.


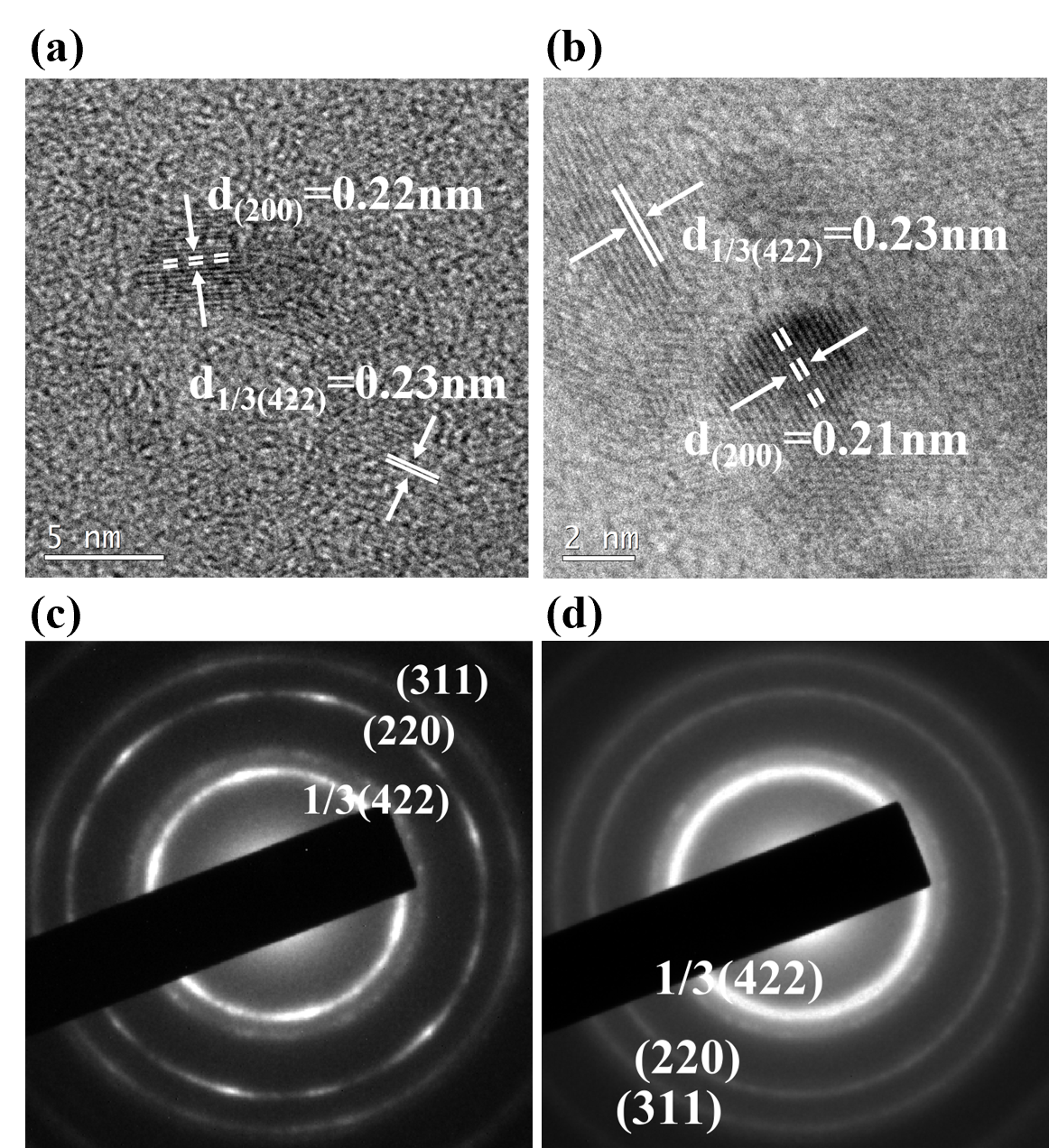


**Figure S5.** (a-b) HRTEM images of CoO_x_/Rh metallene and NiO_x_/Rh metallene. (c-d) SAED selected electron diffraction image of CoO_x_/Rh metallene and NiO_x_/Rh metallene.

In Figure S5a. the solid line with 0.23 nm facet spacing is attributed to the 1/3(422) crystal plane of Rh, and the dashed line with 0.22 nm facet spacing is attributed to the (200) crystal plane of CoO. In Fig. S5b, the solid line with 0.23 nm facet spacing is attributed to the 1/3(422) crystal plane of Rh, and the dashed line with 0.21 nm facet spacing is attributed to the (200) crystal plane of NiO.


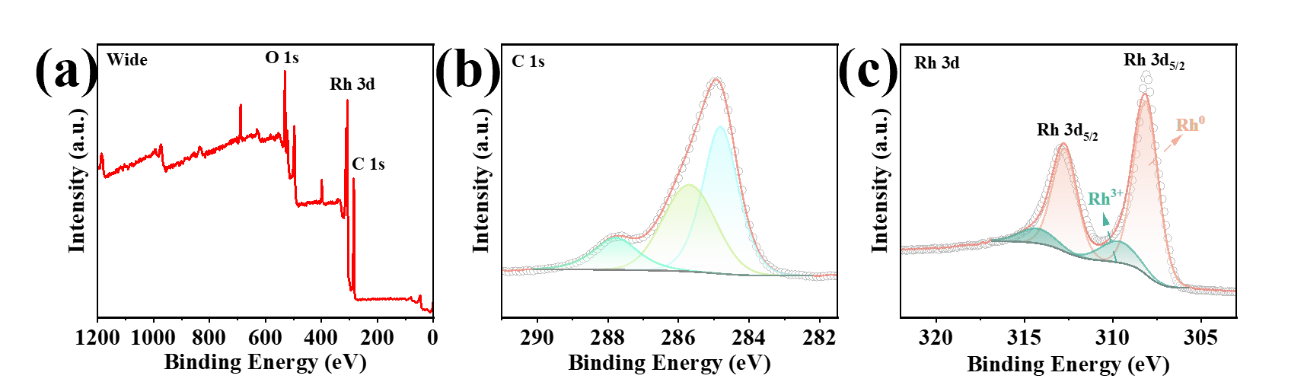


**Figure S6.** XPS spectra of Rh metallene: (a) survey spectrum, and high-resolution spectra of (b) C 1s and (c) Rh 3d.


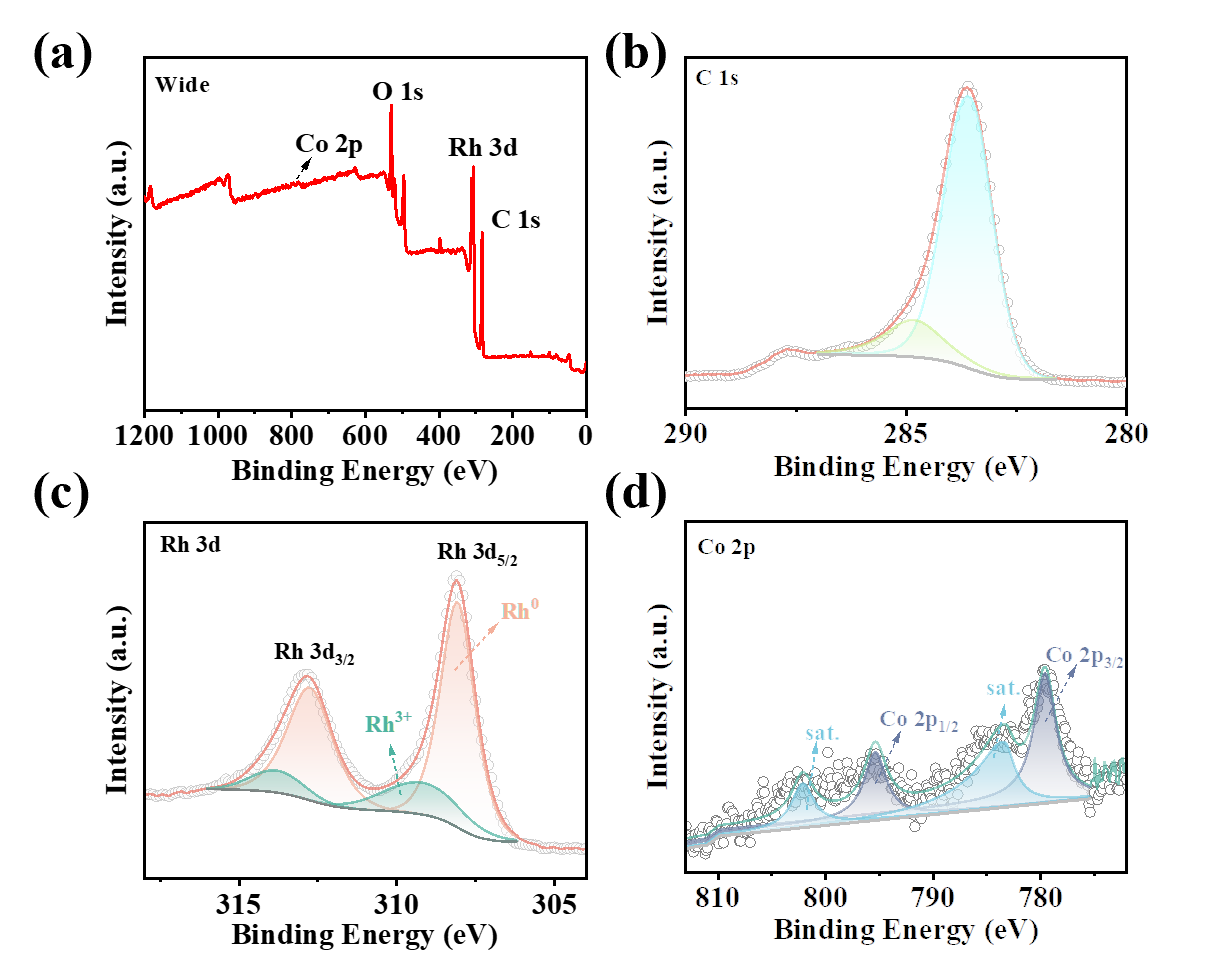


**Figure S7.** (a) XPS spectra of the CoO_x_/Rh metallene: (a) survey spectrum, and high-resolution spectra of (b) C 1s, (c) Rh 3d, and (d) Co 2p.


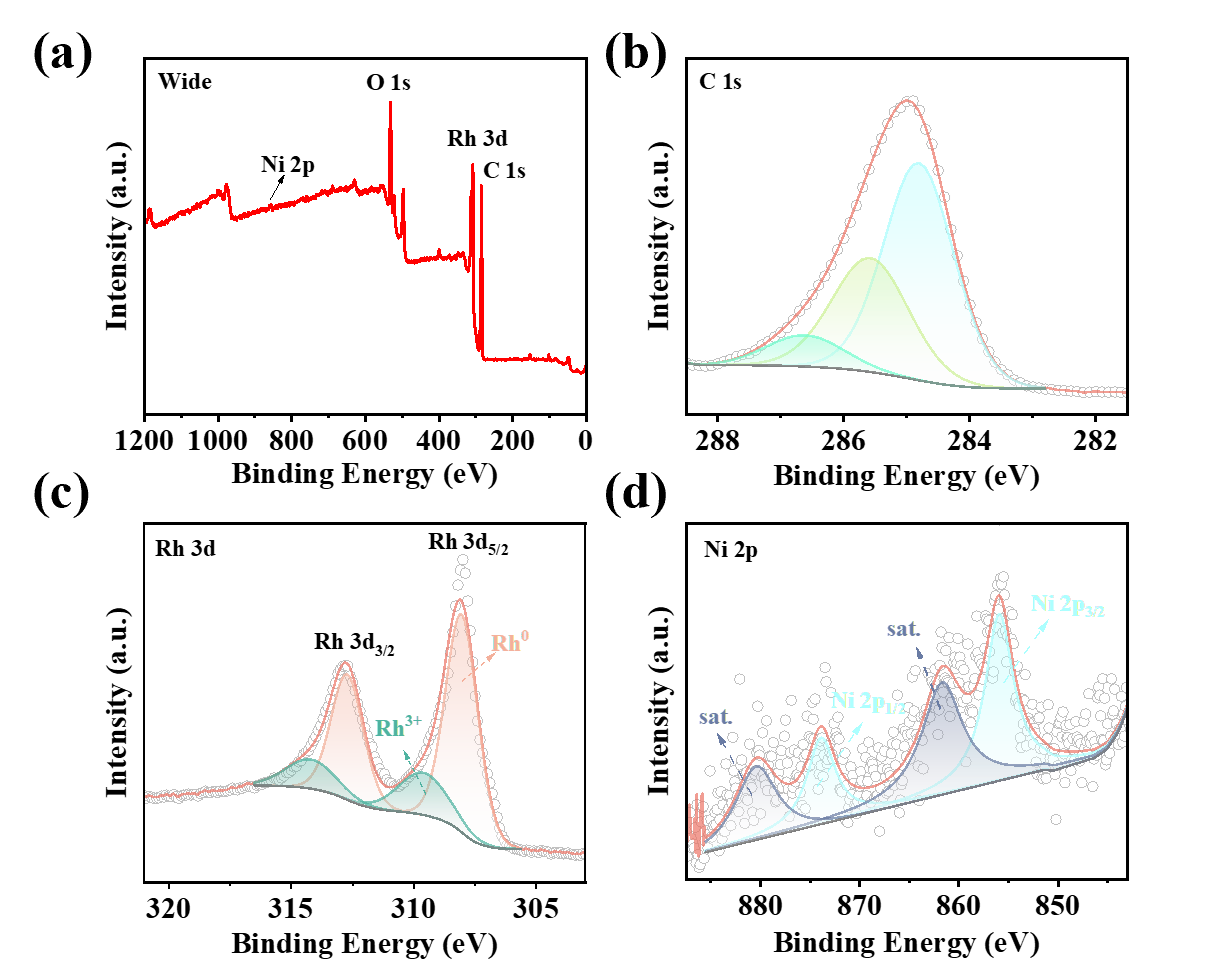


**Figure S8.** XPS spectra of the NiO_x_/Rh metallene: (a) survey spectrum, and high-resolution spectra of (b) C 1s, (c) Rh 3d, and (d) Ni 2p.


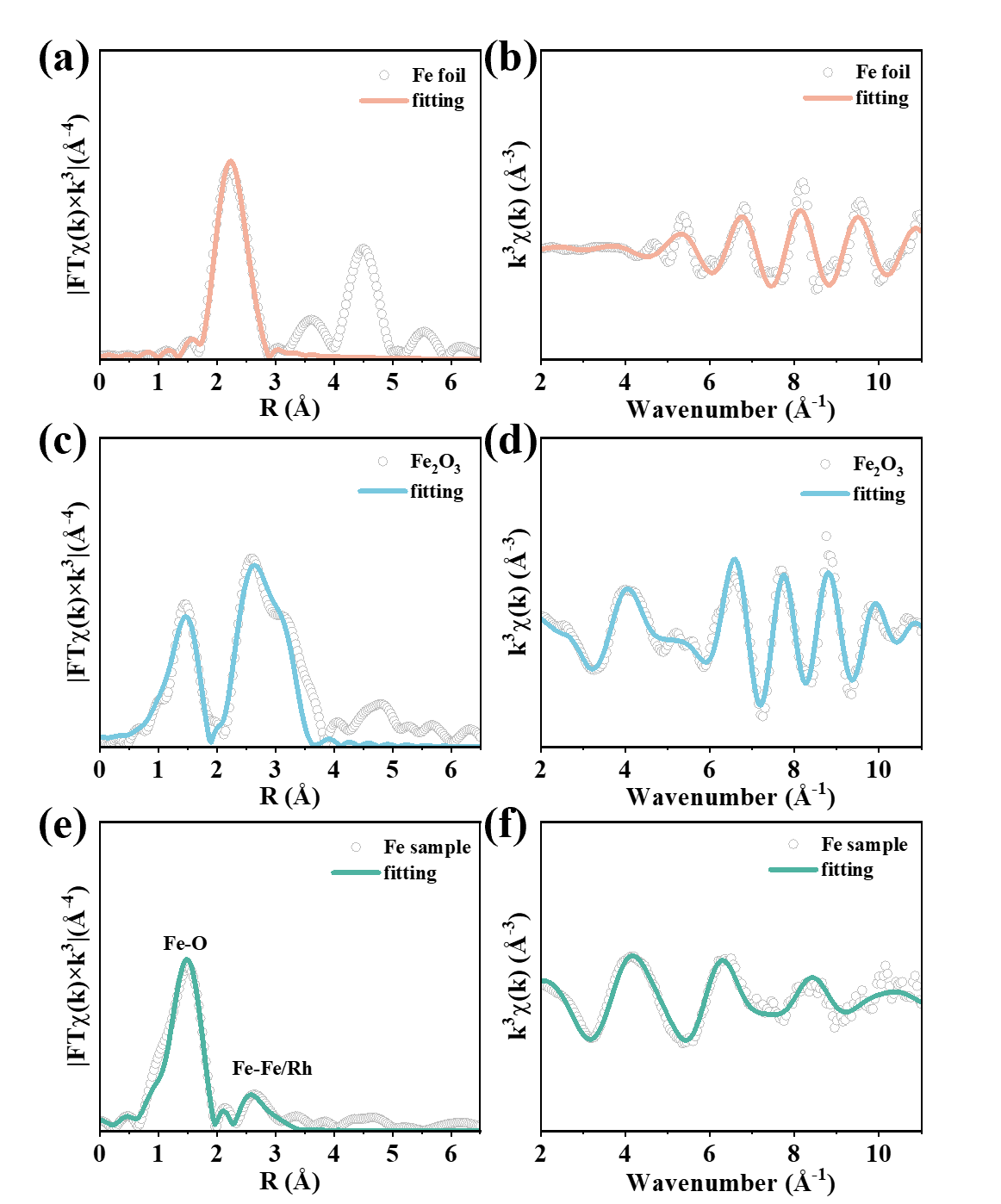


**Figure S9.** The R-space and K-space FT-EXAFS fitting results of the Fe K-edge for (a) Fe foil, (b) Fe_2_O_3_ and (c) FeO_x_/Rh metallene.


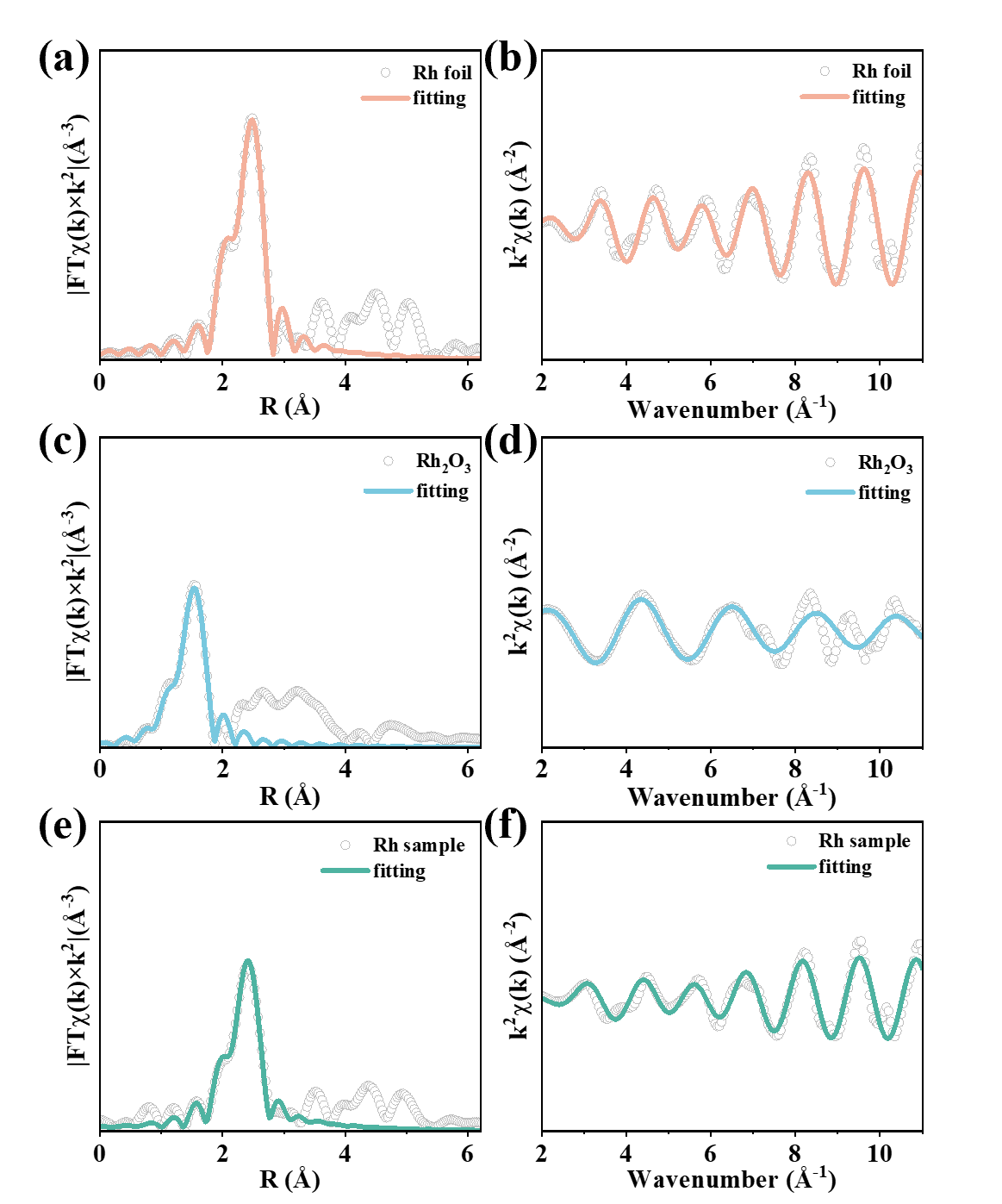


**Figure S10.** The R-space and K-space FT-EXAFS fitting results of the Rh K-edge for (a) Rh foil, (b) Rh_2_O_3_ and (c) FeO_x_/Rh metallene.


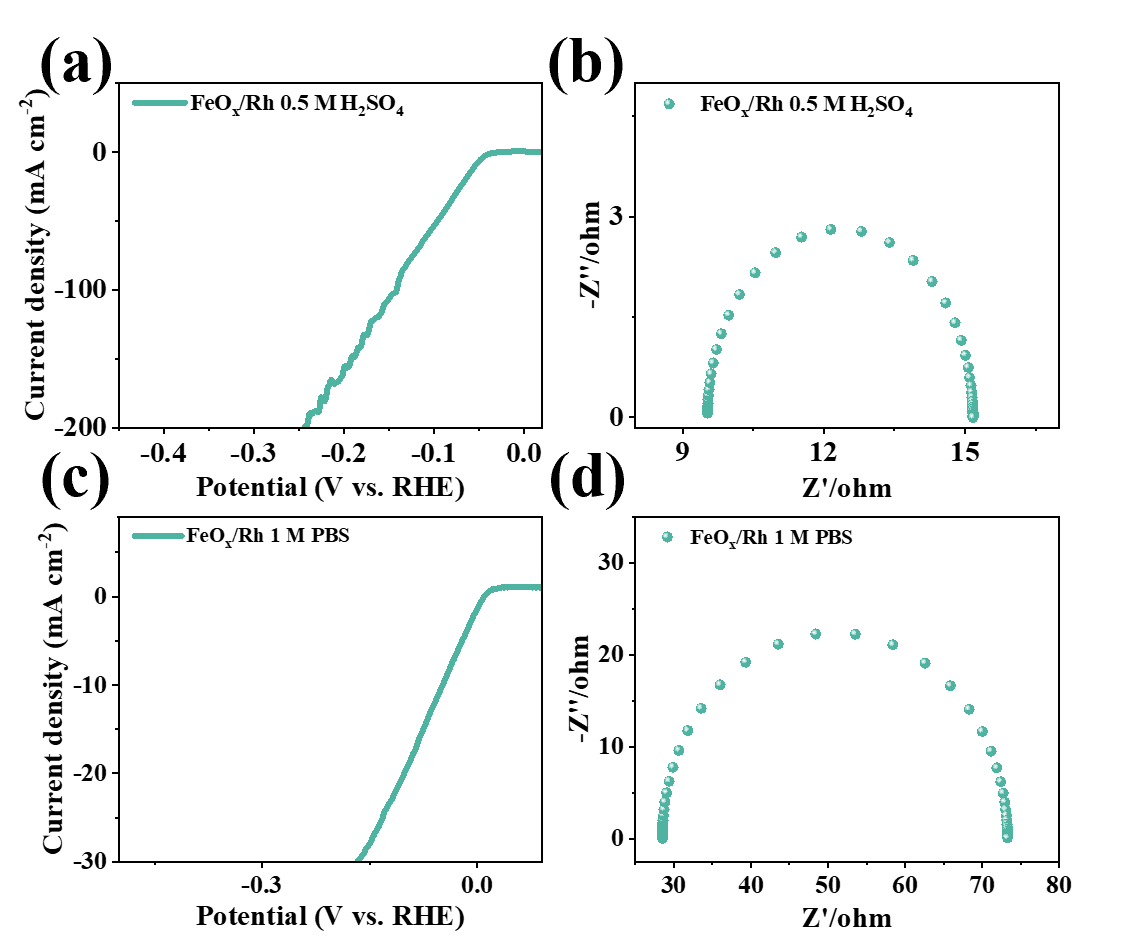


**Figure S11.** Electrocatalytic performance of FeO_x_/Rh metallene in acidic and neutral electrolytes; (a-b) LSV and EIS spectra in 0.5 M H₂SO₄ electrolyte; (c-d) LSV and EIS spectra in 1 M PBS electrolyte.


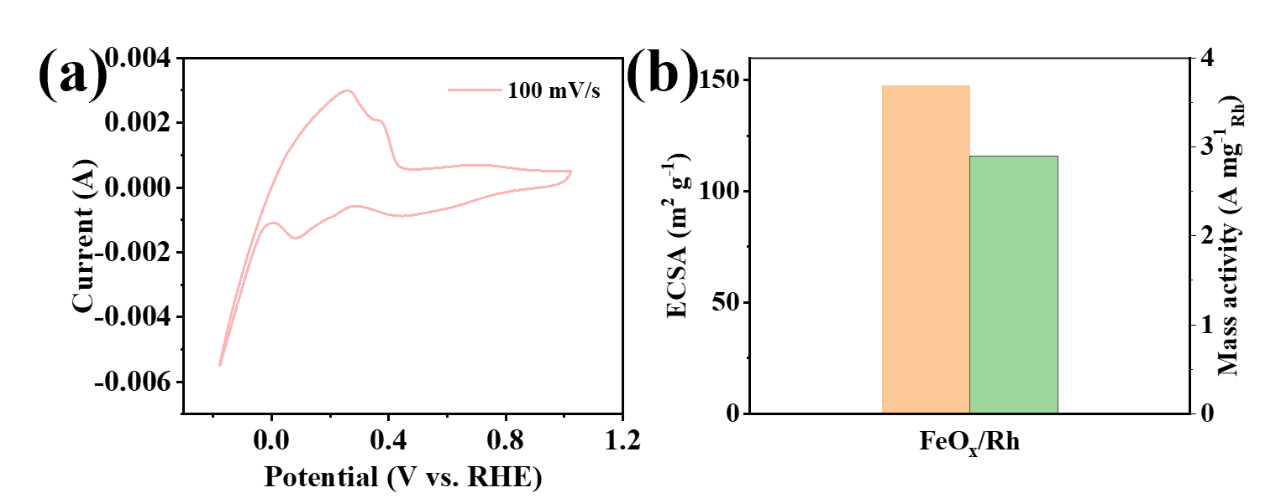


**Figure S12.** (a) CV plot of FeO_x_/Rh at 100 mV/s, (b) ECSA value ( measured by H_upd_ method) and mass activity (η=50 mV) bar graph.


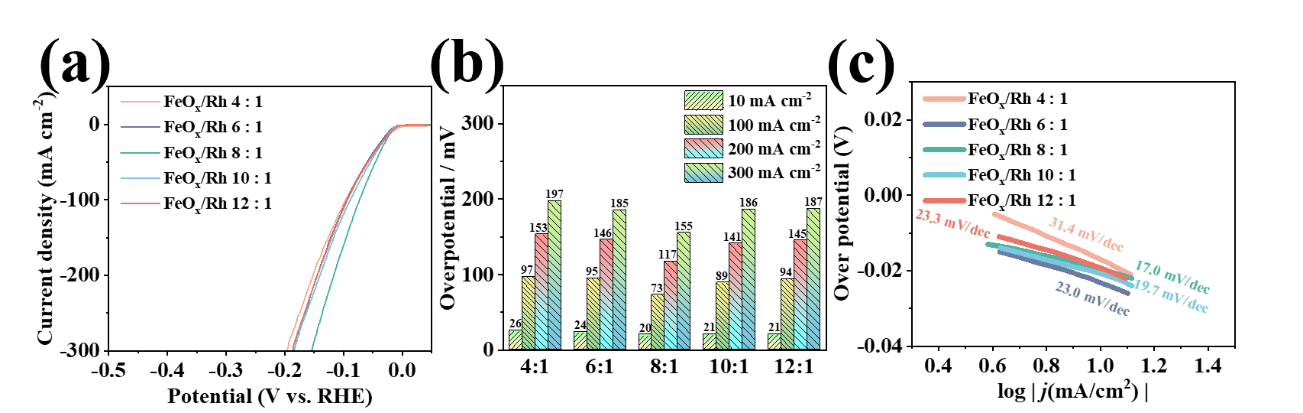


**Figure S13.** HER performance of FeO_x_/Rh metallene in 1 M KOH electrolytes (a) linear sweep voltammetry spectra. (b) Comparison of overpotentials at 10, 100, 200, and 300 mA cm^-2^ current density. (c) Tafel slope plots.


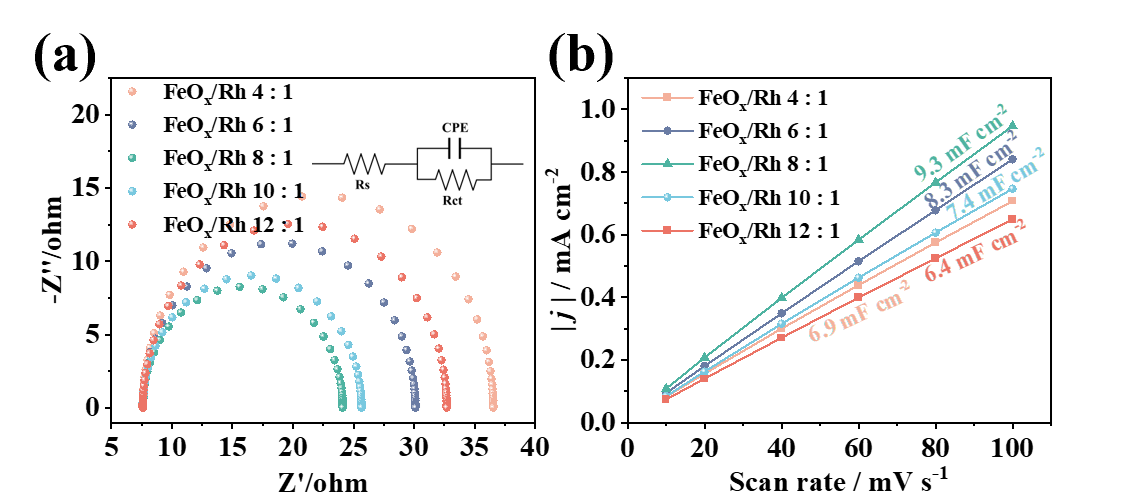


**Figure S14.** HER performance of FeO_x_/Rh metallene in 1 M KOH electrolytes (a) EIS impedance spectra (inset shows equivalent circuit diagram). (b) double layer capacitance (C_dl_) plot.


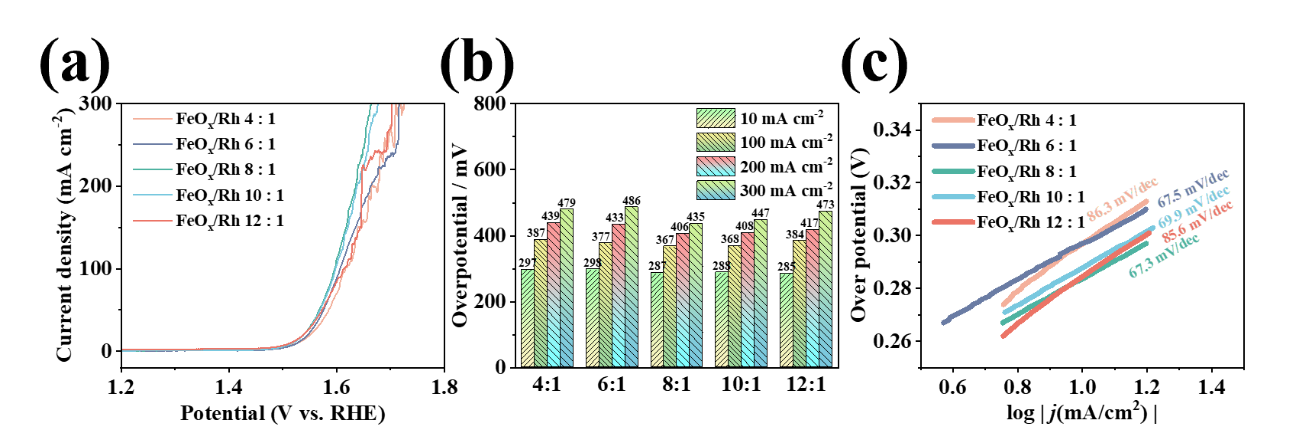


**Figure S15.** OER performance of FeO_x_/Rh metallene in 1 M KOH electrolytes (a) linear sweep voltammetry spectra. (b) Comparison of overpotentials at 10, 100, 200, and 300 mA cm^-2^ current density. (c) Tafel slope plot.


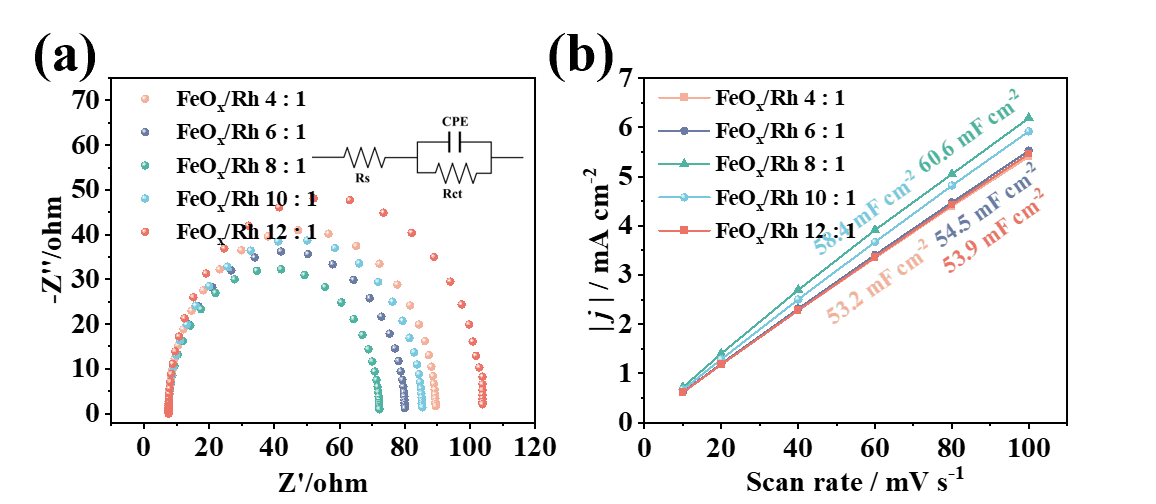


**Figure S16.** OER performance of FeO_x_/Rh metallene in 1 M KOH electrolytes (a) EIS impedance spectra (inset shows equivalent circuit diagram). (b) C_dl_ plot.


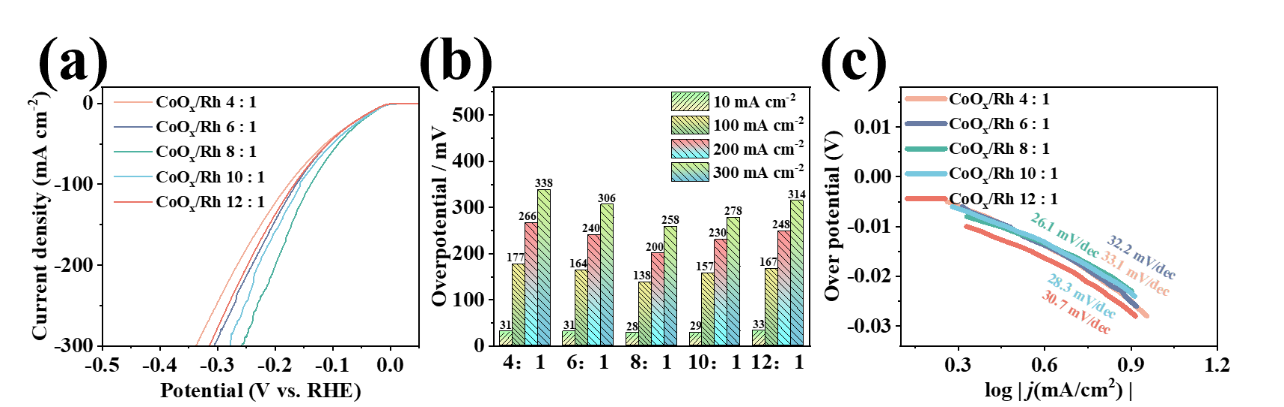


**Figure S17.** HER performance of CoO_x_/Rh metallene in 1 M KOH electrolytes (a) linear sweep voltammetry spectra. (b) Comparison of overpotentials at 10, 100, 200, and 300 mA cm^-2^ current density. (c) Tafel slope plots.


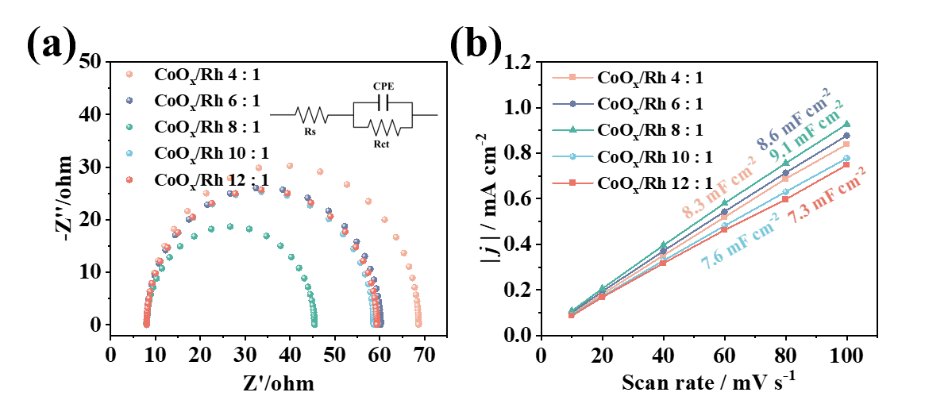


**Figure S18.** HER performance of CoO_x_/Rh metallene in 1 M KOH electrolytes (a) EIS impedance spectra (inset shows equivalent circuit diagram). (b) C_dl_ plot.


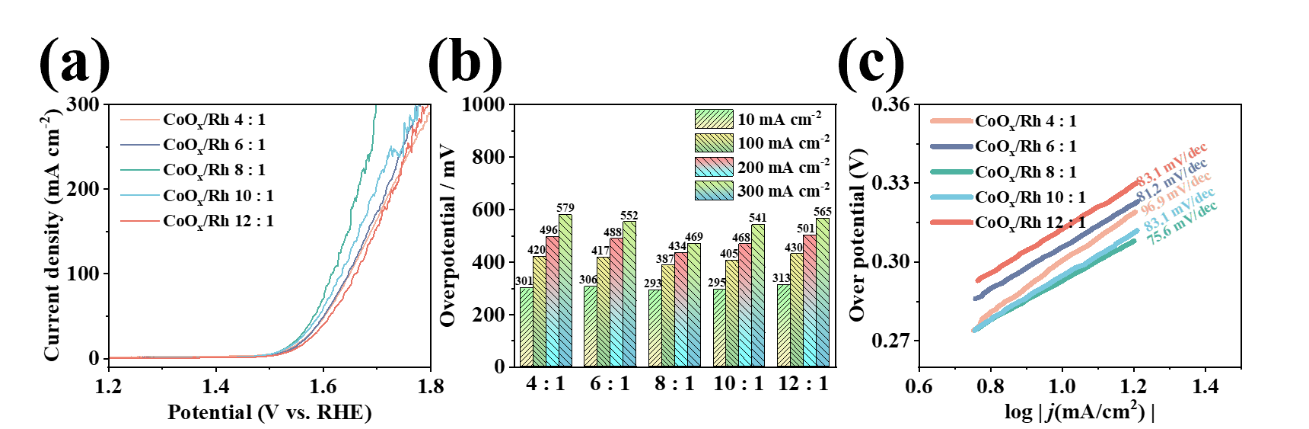


**Figure S19.** OER performance of CoO_x_/Rh metallene in 1 M KOH electrolytes (a) linear sweep voltammetry spectra. (b) Comparison of overpotentials at 10, 100, 200, and 300 mA cm^-2^ current density. (c) Tafel slope plot.


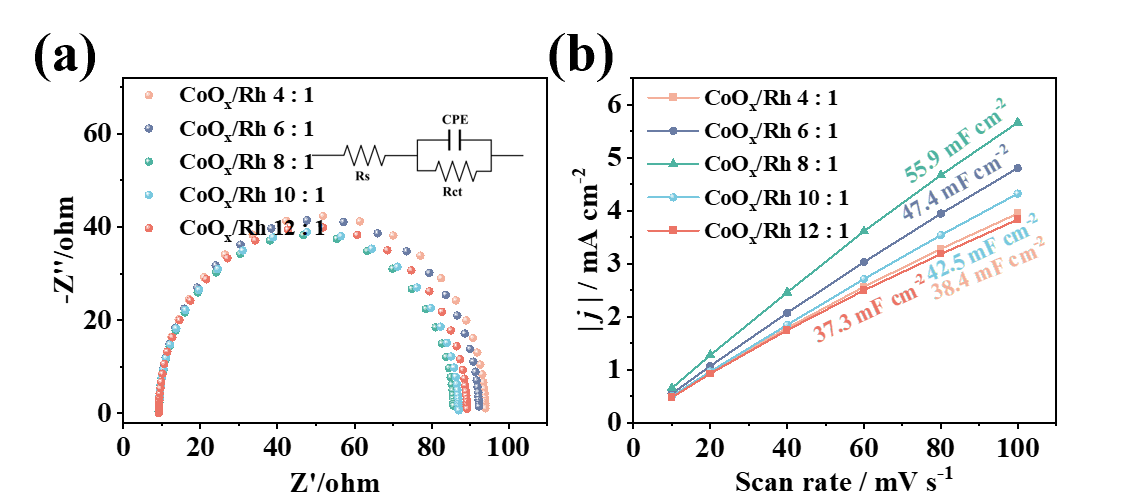


**Figure S20.** OER performance of CoO_x_/Rh metallene in 1 M KOH electrolytes (a) EIS impedance spectra (inset shows equivalent circuit diagram). (b) C_dl_ plot.


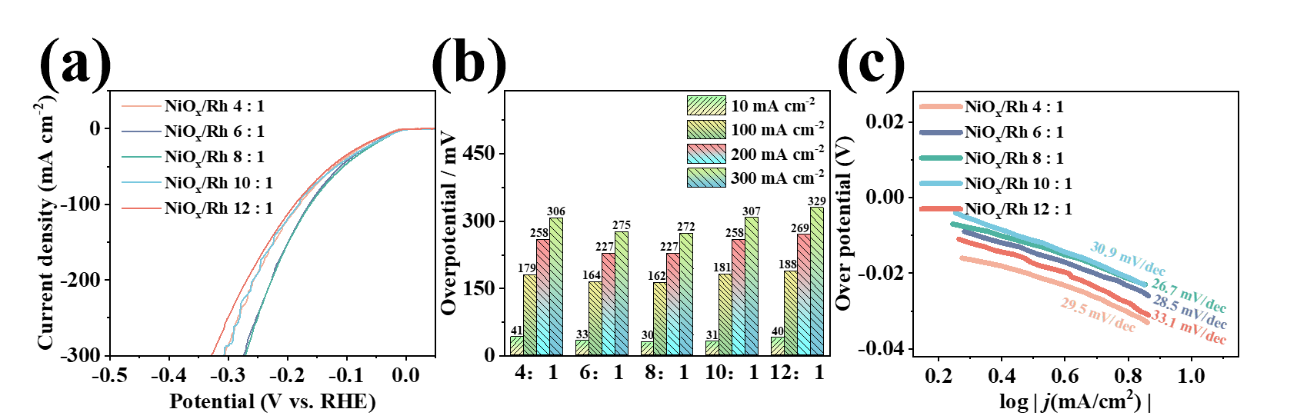


**Figure S21.** HER performance of NiO_x_/Rh metallene in 1 M KOH electrolytes (a) linear sweep voltammetry spectra. (b) Comparison of overpotentials at 10, 100, 200, and 300 mA cm^-2^ current density. (c) Tafel slope plots.


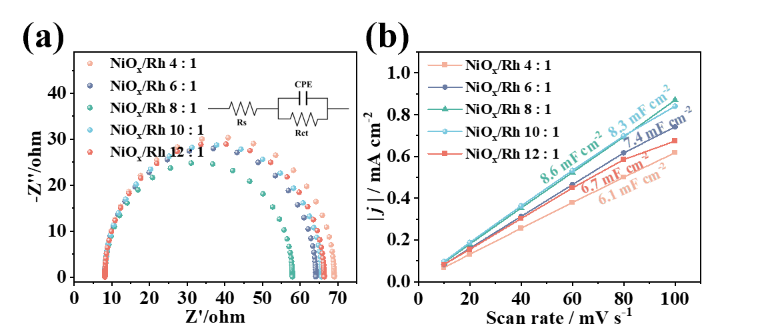


**Figure S22.** HER performance of NiO_x_/Rh metallene in 1 M KOH electrolytes (a) EIS impedance spectra (inset shows equivalent circuit diagram). (b) C_dl_ plot.


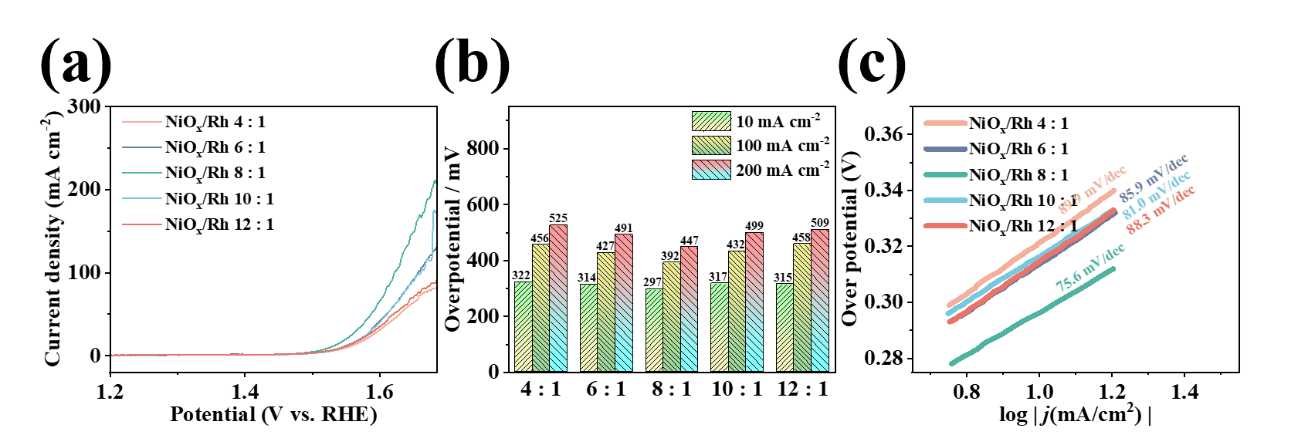


**Figure S23.** OER performance of NiO_x_/Rh metallene in 1 M KOH electrolytes (a) linear sweep voltammetry spectra. (b) Comparison of overpotentials at 10, 100, 200, and 300 mA cm^-2^ current density. (c) Tafel slope plot.


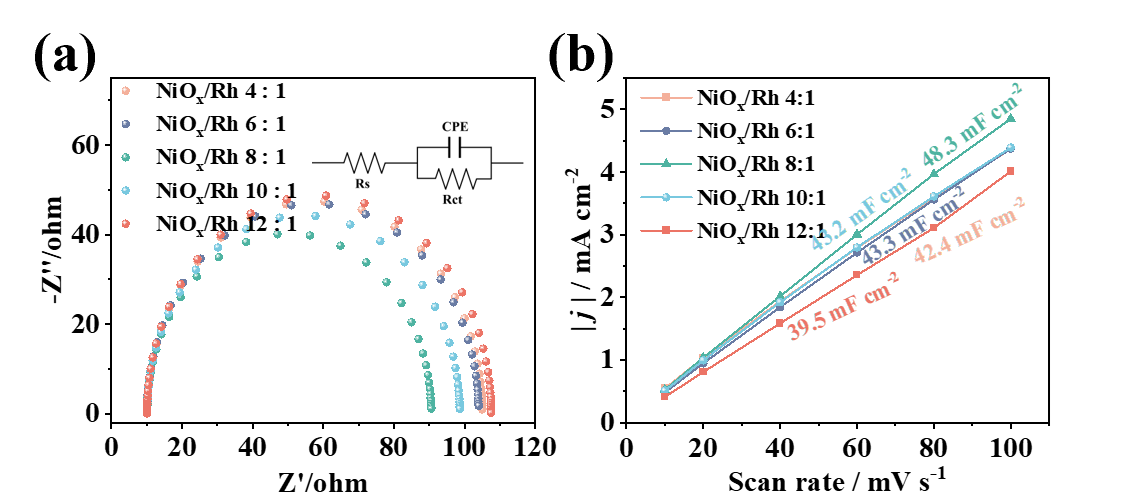


**Figure S24.** OER performance of NiO_x_/Rh metallene in 1 M KOH electrolytes (a) EIS impedance spectra (inset shows equivalent circuit diagram). (b) C_dl_ plot.


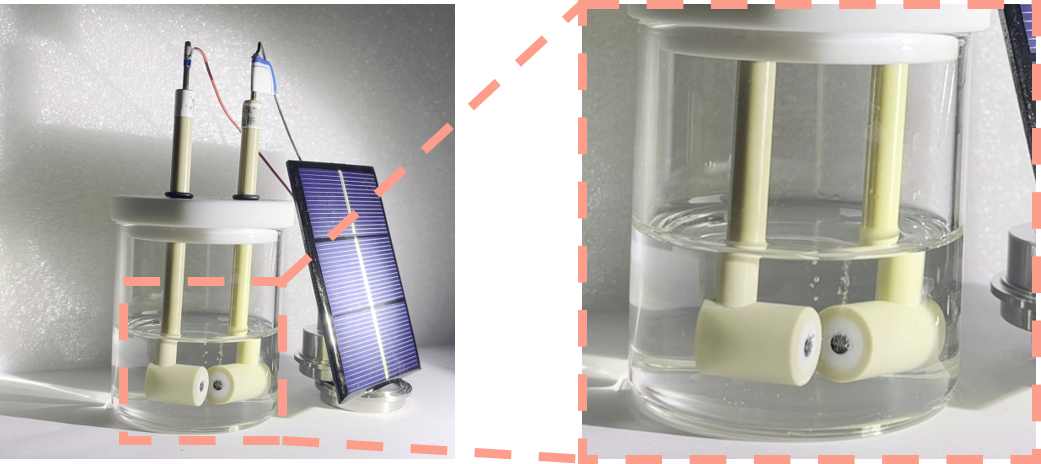


**Figure S25.** Simplified diagram of a solar-powered water electrolysis unit (the inset image is a local enlargement).


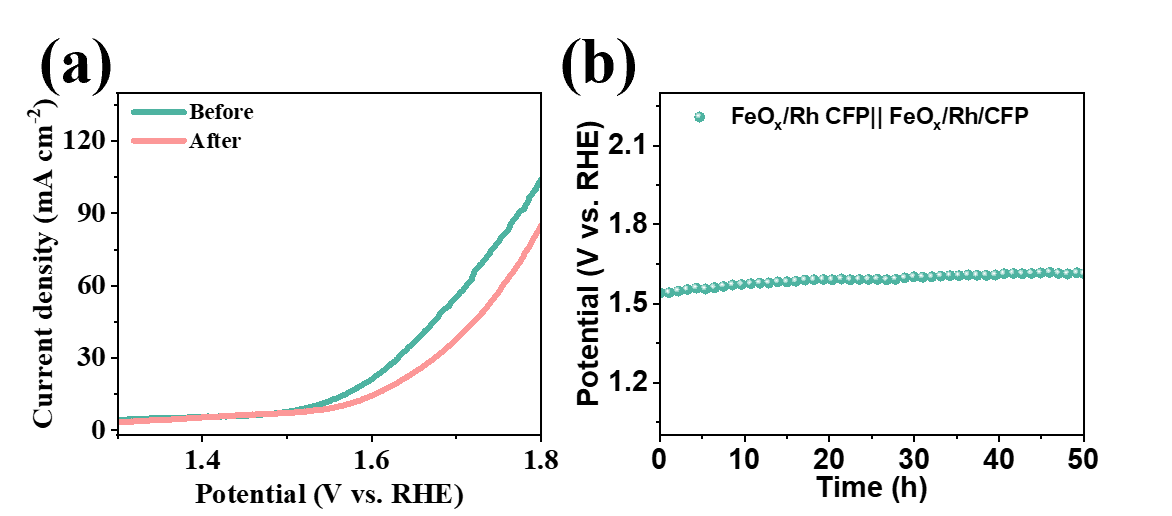


**Figure S26.** **(a)** LSV polarization curves before and after chronopotentiometry testing; (b) chronopotentiometry plot.


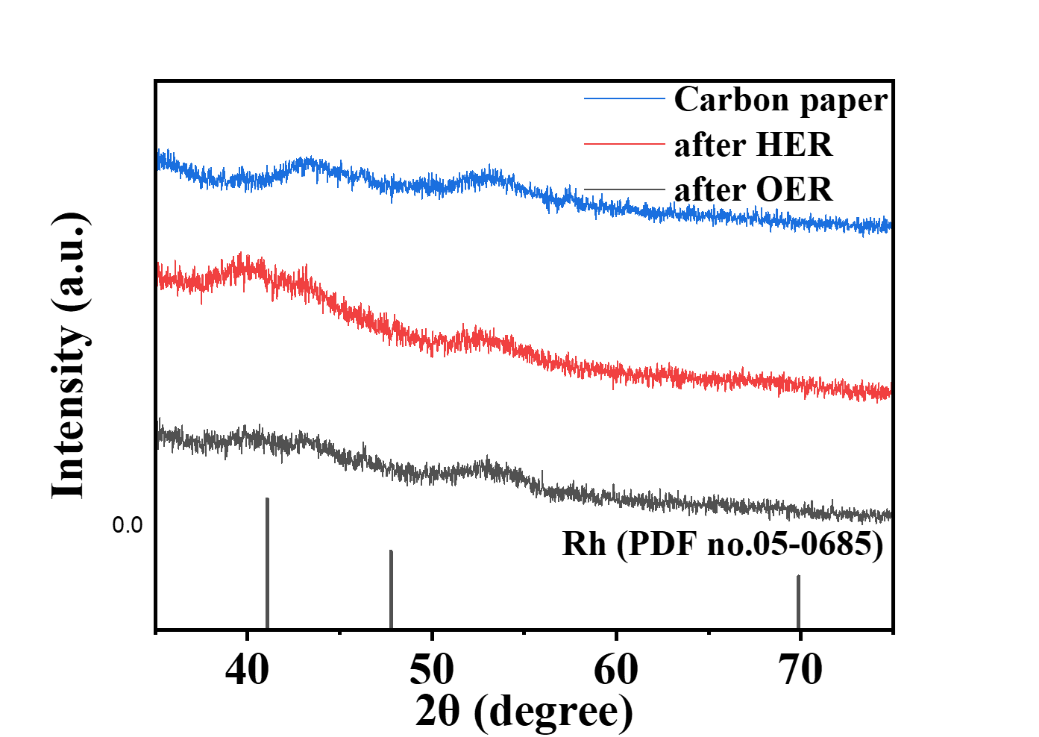


**Figure S27.** XRD spectra of FeO_x_/Rh metallene before and after i-t measurement.


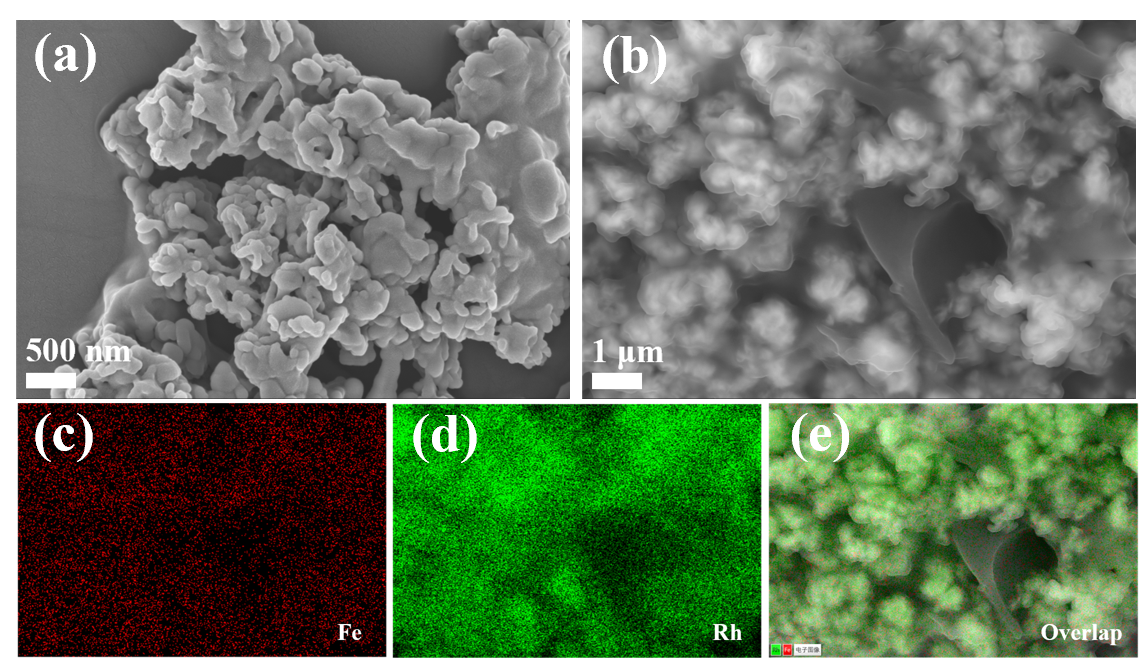


**Figure S28.** Figure FESEM images and corresponding element mapping images of FeO_x_/Rh metallene on carbon paper after i-t measurement (after HER). (a) FESEM. (b-e) Element mapping images.

The agglomeration of metallene morphology might be caused by the addition of nafion during testing. This phenomenon can also be observed in samples prior to stability testing.


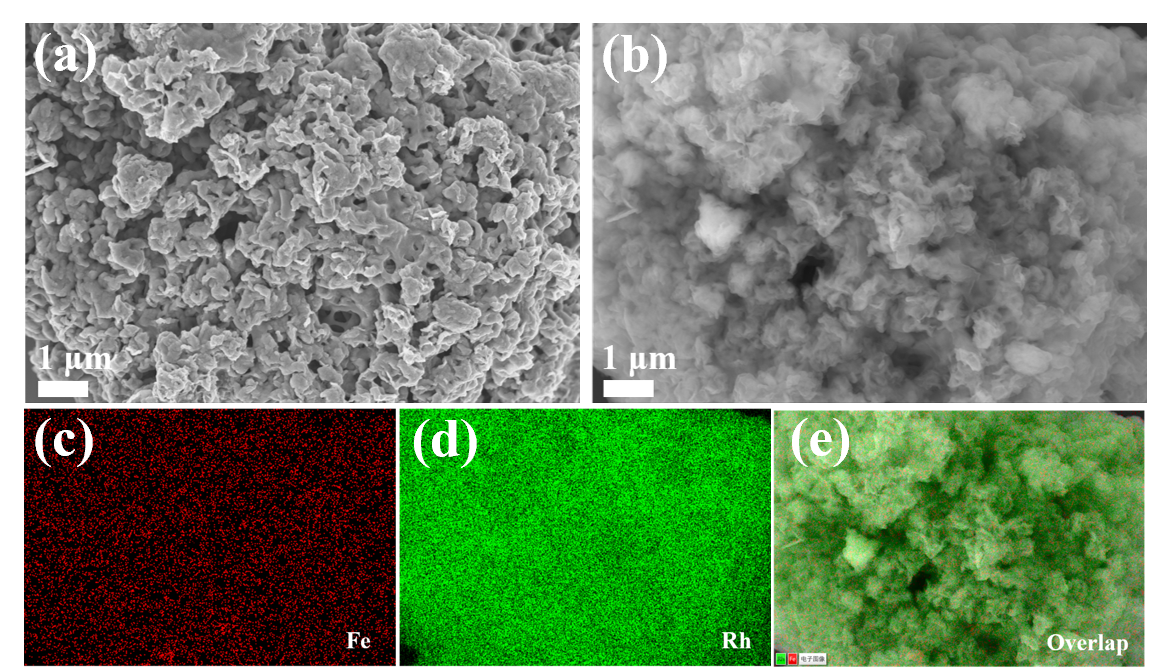


**Figure S29.** Figure FESEM images and corresponding element mapping images of FeO_x_/Rh metallene on carbon paper after i-t measurement (after OER). (a) FESEM. (b-e) Element mapping images.


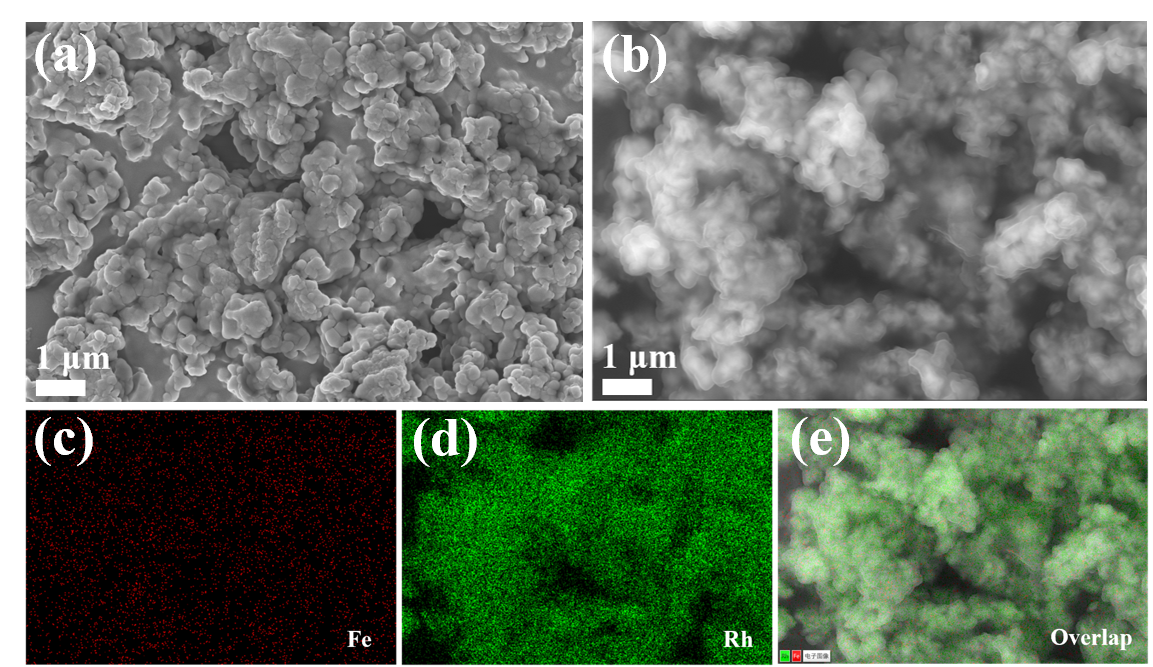


**Figure S30.** Figure FESEM images and corresponding element mapping images of FeO_x_/Rh metallene on carbon paper after i-t measurement (before test). (a) FESEM. (b-e) Element mapping images.


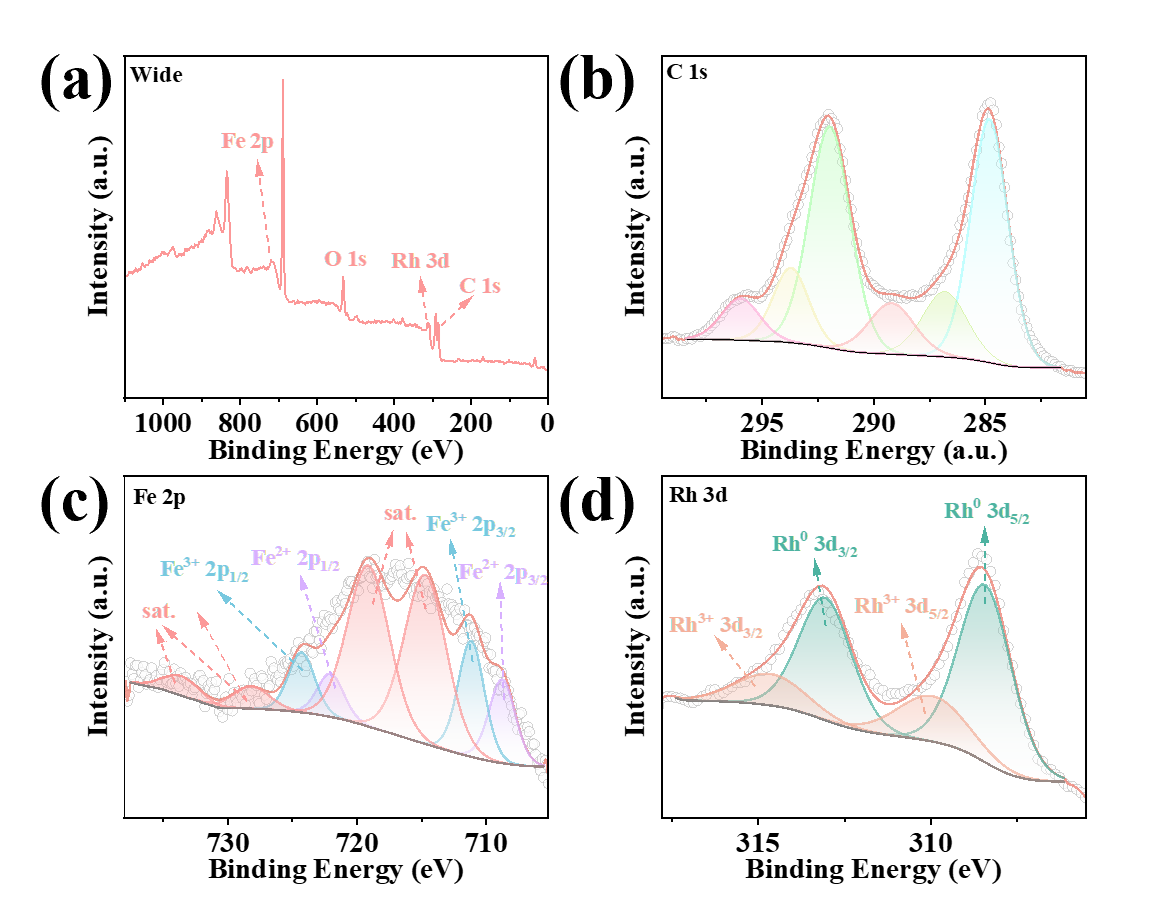


**Figure S31.** XPS spectrum of FeO_x_/Rh metallene loaded on carbon paper after durability testing. (a) survey spectrum, high-resolution spectra of (b) C 1s, (c) Fe 2p and (d) Rh 3d.

XPS analysis was conducted to investigate the chemical states of FeO_x_/Rh metalene after durability testing. As shown in the figure S31, the peak at 708.75 eV corresponds to Fe^2+^ 2p_3/2_, while the peak at 711.13 eV corresponds to Fe^3+^ 2p_3/2_, indicating a decreased binding energy. The peak at 308.5 eV corresponds to Rh^0^ 3d_5/2_, while the peak at 310.13 eV corresponds to Rh^3+^ 3d_5/2_, indicating an increased binding energy. The valence states of Fe and Rh elements remained consistent with those before cycling tests, demonstrating catalyst stability.


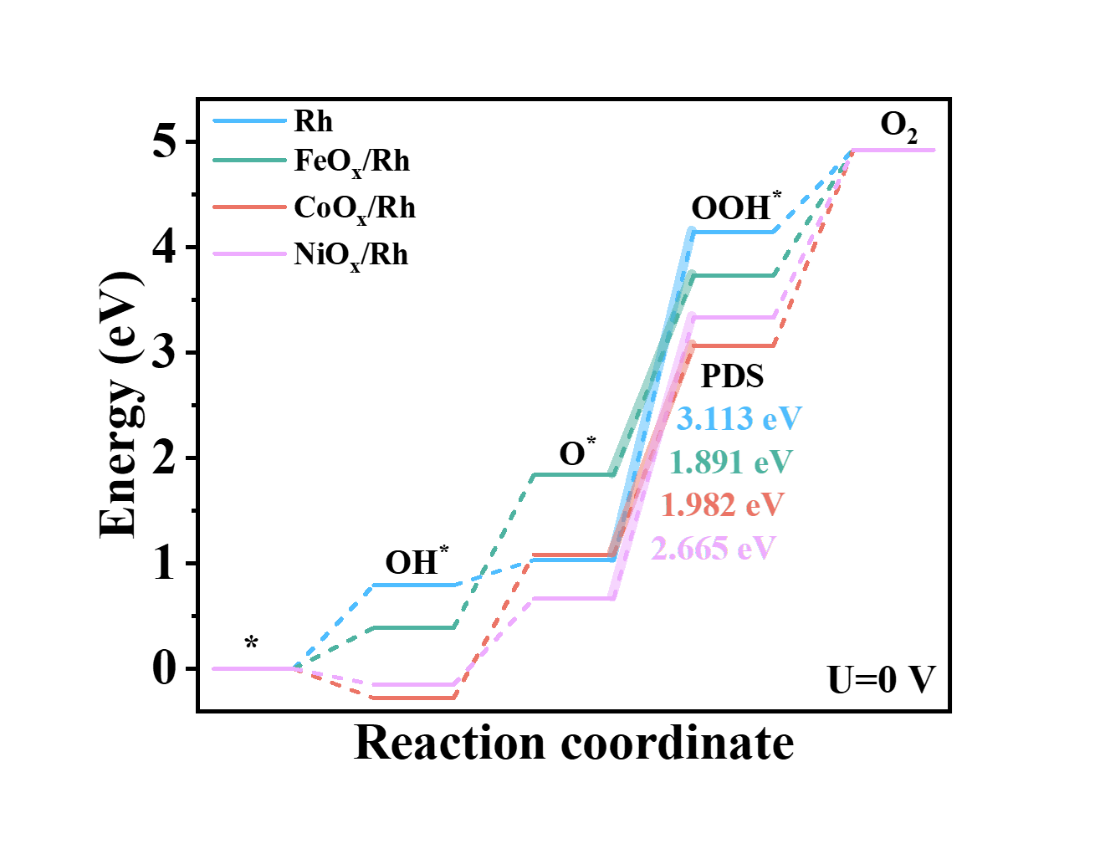


**Figure S32.** (a) Calculated free energy step diagram relative to RHE at potential U = 0 V.


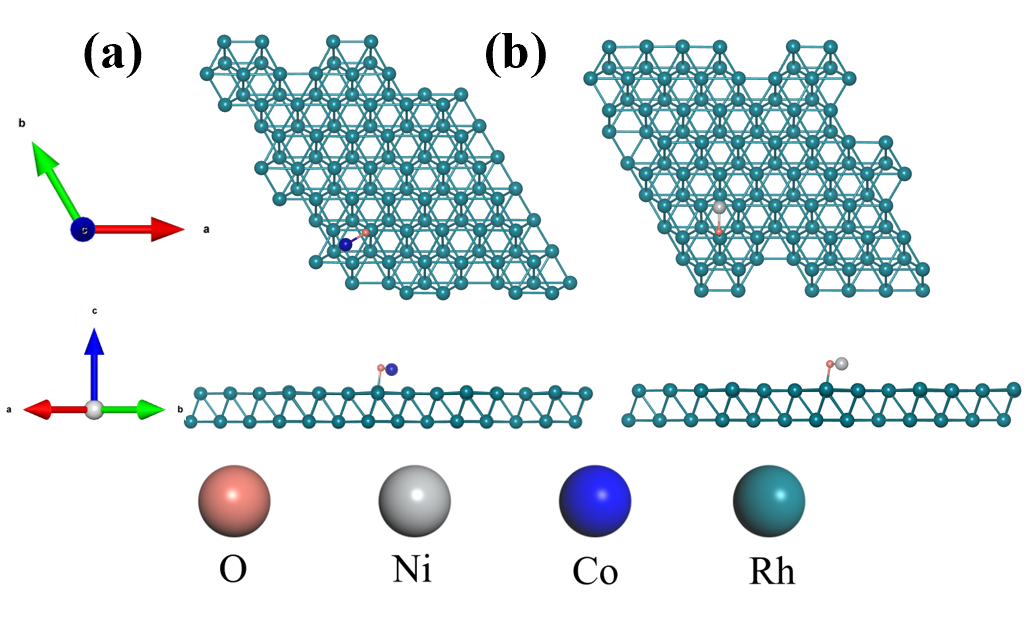


**Figure S33.** Structural optimized configurations of CoO_x_/Rh metallene and NiO_x_/Rh metallene.


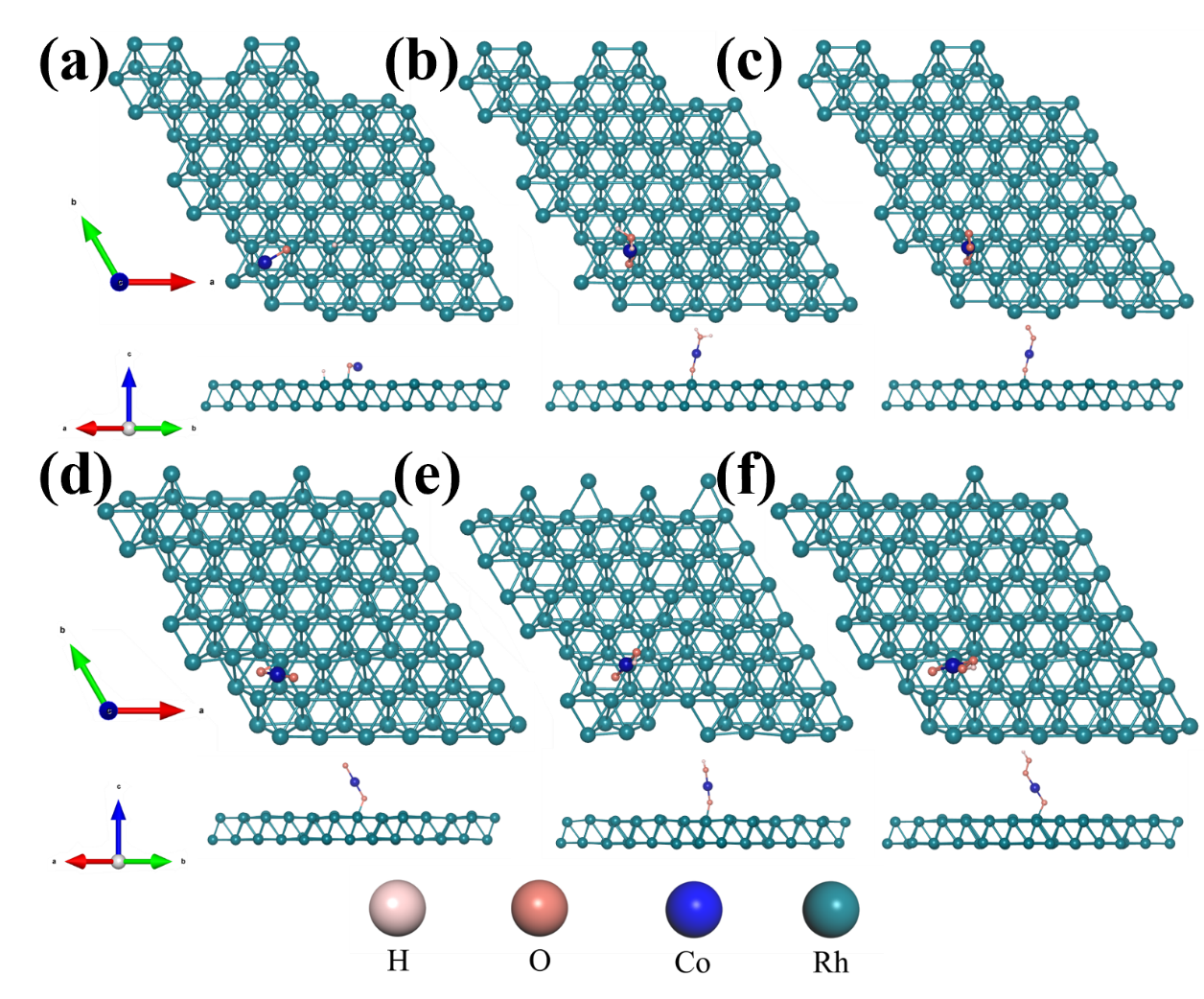


**Figure S34.** (a-c) Configurations of H^*^、H_2_O^*^ and O_2_^*^ intermediates adsorbed on CoO_x_/Rh metallene surface (H^*^ intermediate adsorbs on Rh site). (d-f) Configurations of OER intermediates adsorbed on CoO_x_/Rh metallene surface.


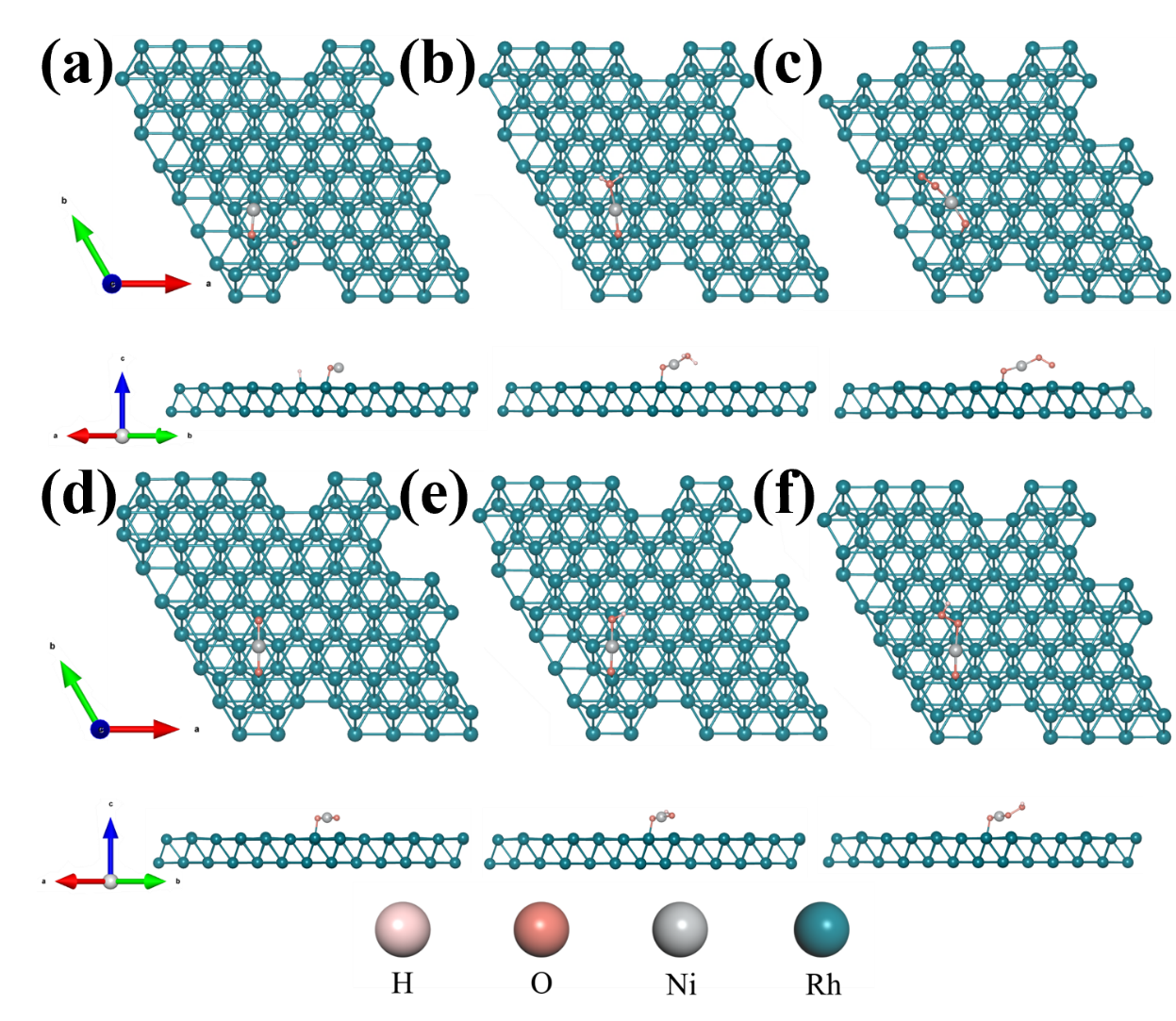


**Figure S35.** (a-c) Configurations of H^*^、H_2_O^*^ and O_2_^*^ intermediates adsorbed on NiO_x_/Rh metallene surface (H^*^ intermediate adsorbs on Rh site). (d-f) Configurations of OER intermediates adsorbed on NiO_x_/Rh metallene surface.


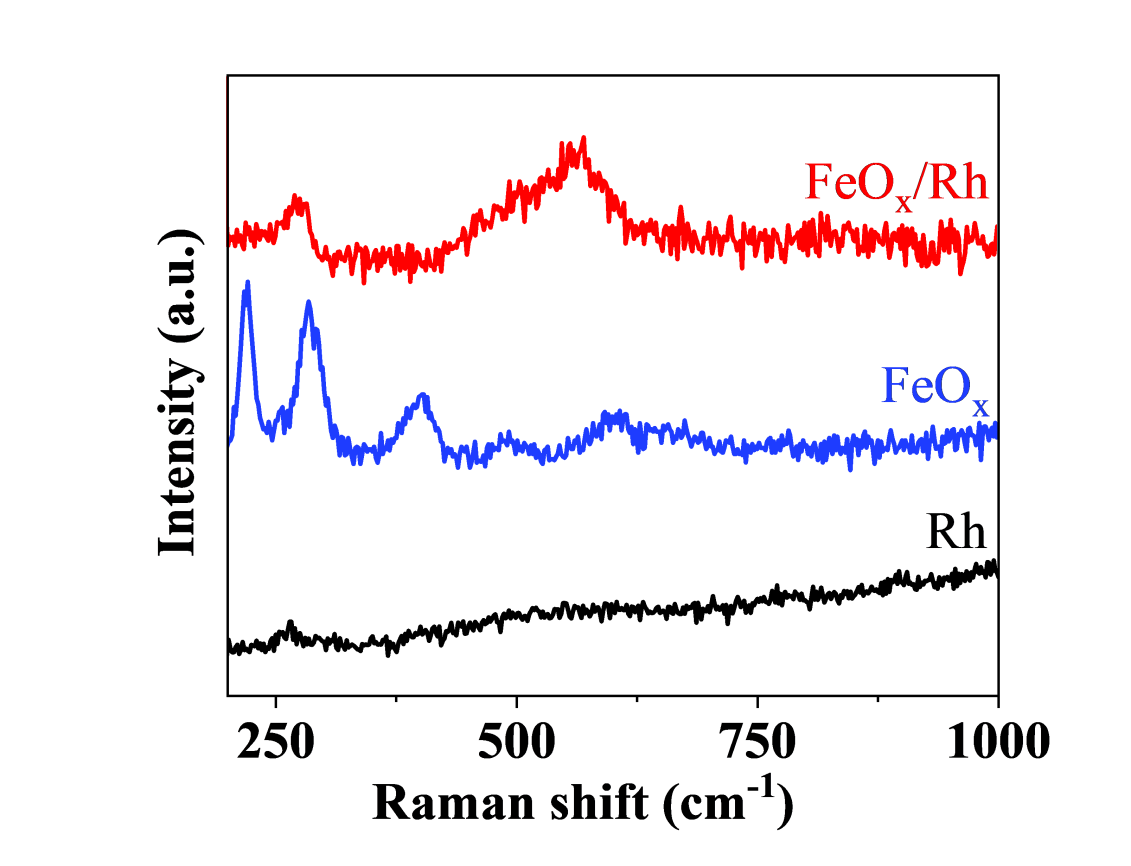


**Figure S36.** Raman spectra of FeO_x_/Rh, pure FeO_x_ and Rh metallene.


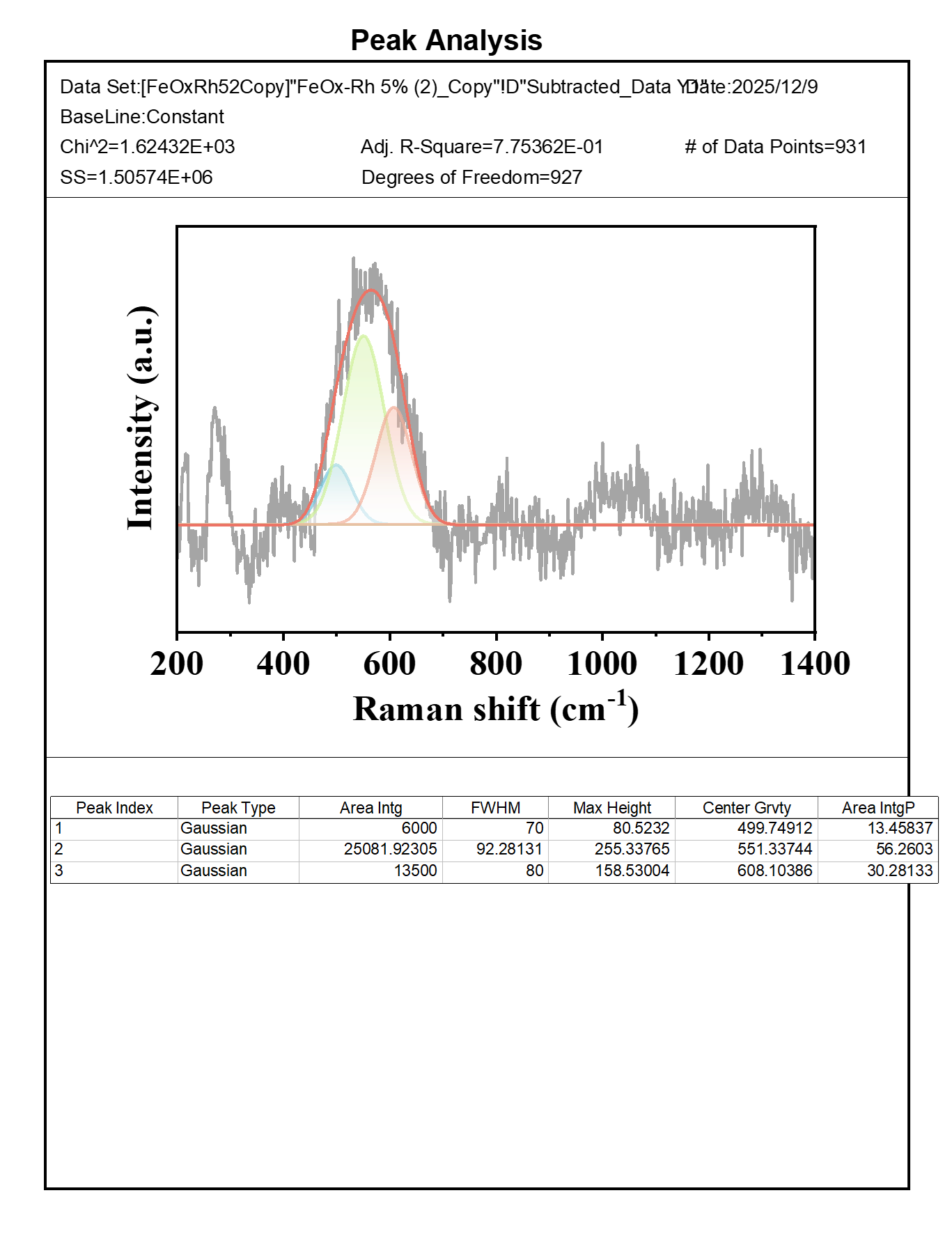


**Figure S37.** Lorentzian Deconvolution of the Raman Spectrum for FeO_x_/Rh at ~567 cm^-1^.


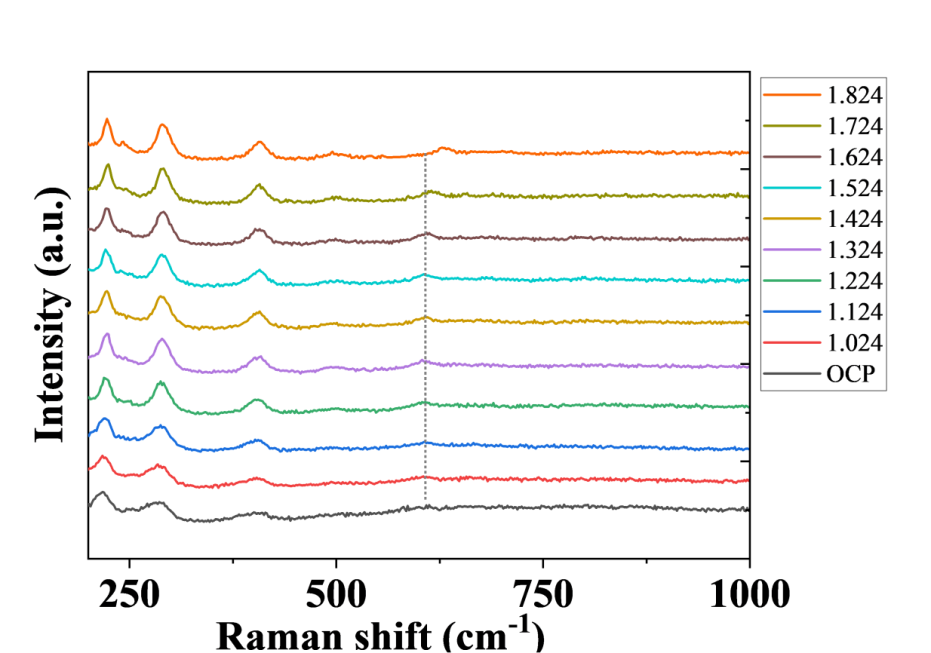


**Figure S38.** In situ Raman spectra of FeO_x_.


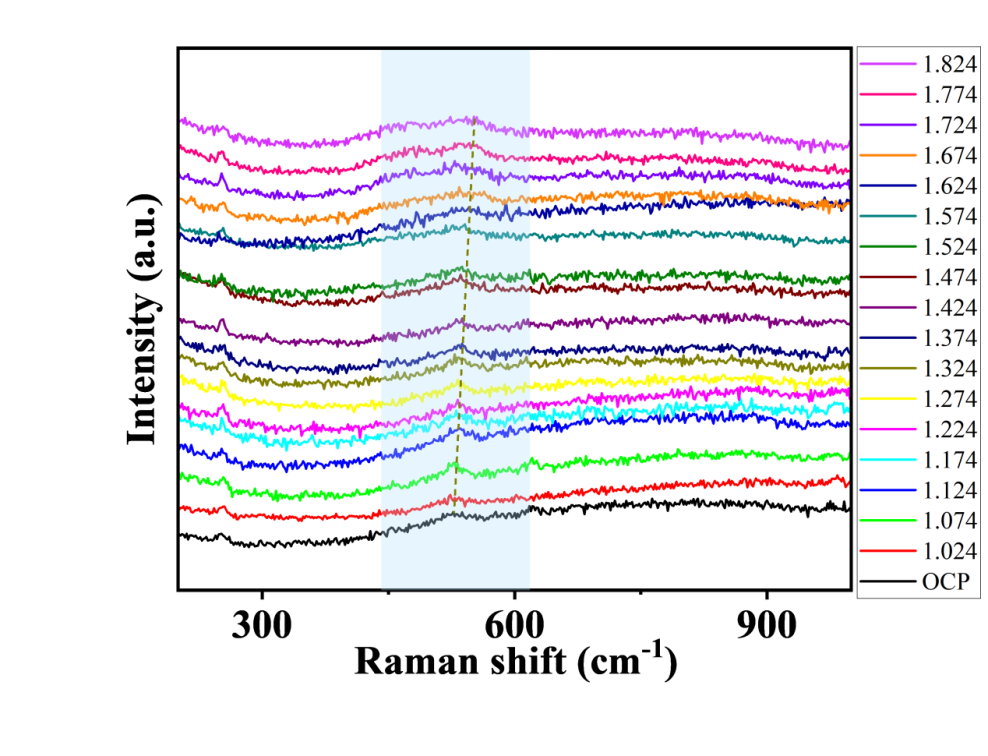


**Figure S39.** In situ Raman spectra of FeO_x_/Rh.


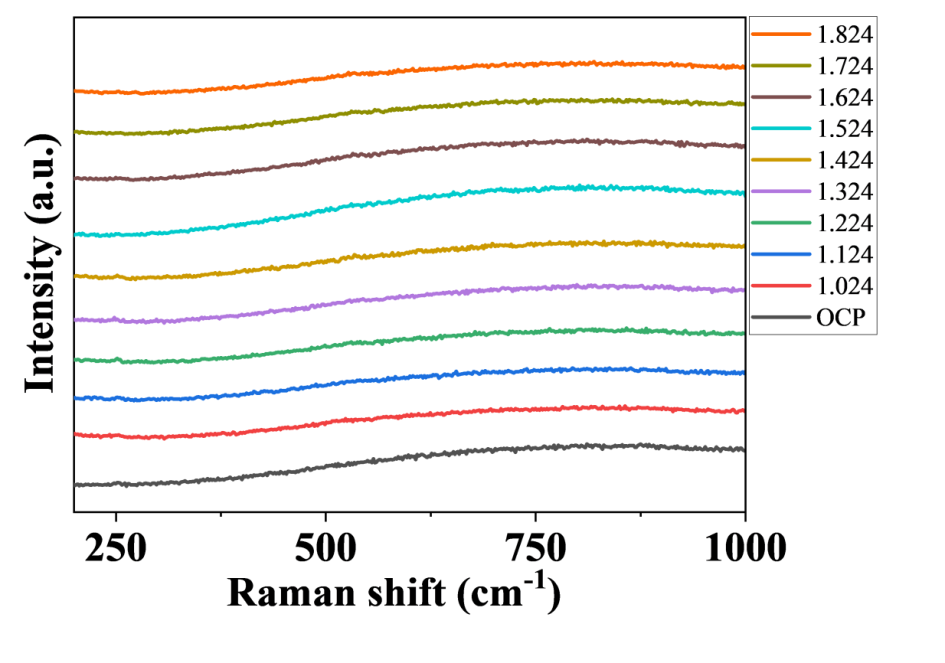


**Figure S40.** In situ Raman spectra of Rh metallene.

**Table S1.** EXAFS fitting parameters at the Fe K–edge for various samples.

| Sample | Shell | *CN^a^* | *R*(Å)*^b^* | *σ*^2^(Å^2^)*^c^* | Δ*E*_0_(eV)*^d^* | *R* factor |
| --- | --- | --- | --- | --- | --- | --- |
| Fe foil | Fe-Fe | 8* | 2.49±0.01 | 0.0068 | 9.0 | 0.0086 |
|  | Fe-Fe | 6* | 2.81±0.01 | 0.0092 | 4.9 |  |
| Fe_2_O_3_ | Fe-O | 6* | 1.96±0.01 | 0.0110 | -2.8 | 0.0189 |
|  | Fe-Fe | 8* | 2.97±0.01 | 0.0103 | 2.5 |  |
|  | Fe-Fe | 4* | 3.41±0.01 | 0.0058 | 12.7 |  |
| **Fe sample** | **Fe-O** | **6.0±0.3** | **1.98±0.01** | **0.0104** | **-0.8** | **0.0135** |
|  | **Fe-Fe/Rh** | **2.1±1.0** | **3.10±0.01** | **0.0150** | **4.1** |  |

*^a^CN*, coordination number; *^b^R*, distance between absorber and backscatter atoms; *^c^σ*^2^, Debye-Waller factor to account for both thermal and structural disorders; *^d^ΔE*_0_, inner potential correction; *R* factor indicates the goodness of the fit; S_0_^2^ was fixed to 0.859 according to the experimental EXAFS fit of Fe foil by fixing CN as the known crystallographic value; A reasonable range of EXAFS fitting parameters: 0.600 < *Ѕ*_0_^2^ < 1.000; *CN* > 0; *σ*^2^ > 0 Å^2^; |Δ*E*_0_| < 15 eV; *R* factor < 0.02.

**Table S2.** EXAFS fitting parameters at the Rh K–edge for various samples.

| Sample | Shell | *CN^a^* | *R*(Å)*^b^* | *σ*^2^(Å^2^)*^c^* | Δ*E*_0_(eV)*^d^* | *R* factor |
| --- | --- | --- | --- | --- | --- | --- |
| Rh foil | Rh-Rh | 12* | 2.68±0.01 | 0.0030 | 3.6 | 0.0066 |
| Rh_2_O_3_ | Rh-O | 6* | 2.04±0.01 | 0.0007 | -1.1 | 0.0060 |
| **Rh sample** | **Rh-Rh** | **8.3±0.7** | **2.68±0.01** | **0.0028** | **-4.2** | **0.0045** |

*^a^CN*, coordination number; *^b^R*, distance between absorber and backscatter atoms; *^c^σ*^2^, Debye-Waller factor to account for both thermal and structural disorders; *^d^ΔE*_0_, inner potential correction; *R* factor indicates the goodness of the fit; S_0_^2^ was fixed to 0.795, according to the experimental EXAFS fit of Rh foil by fixing CN as the known crystallographic value; A reasonable range of EXAFS fitting parameters: 0.600 < *Ѕ*_0_^2^ < 1.000; *CN* > 0; *σ*^2^ > 0 Å^2^; |Δ*E*_0_| < 15 eV; *R* factor < 0.02.

**Table S3.** Rh/FeO_x_ metallene ICP-OES test results.

| **Elements** | **m_0_（g)** | **V_0_（mL)** | **C_o_（mg/L)** | **f** | **C_1_（mg/L)** | **C_x_（mg/kg)** | **C（mg/kg)** | **W (%)** |
| --- | --- | --- | --- | --- | --- | --- | --- | --- |
| Fe | 0.0411 | 25 | 4.322 | 10 | 43.225 | 26292.375 | 26688.922 | **2.67%** |
|  |  |  | 4.372 |  | 43.723 | 26595.727 |  |  |
|  |  |  | 4.468 |  | 44.682 | 27178.664 |  |  |
| Rh | 0.0411 | 25 | 1.354 | 1000 | 1353.736 | 823440.371 | 786991.064 | **78.70%** |
|  |  |  | 1.285 |  | 1285.213 | 781760.024 |  |  |
|  |  |  | 1.242 |  | 1242.490 | 755772.798 |  |  |

The data is calculated by the following equation:

$\boldsymbol{Cx}\left( \frac{\boldsymbol{mg}}{\boldsymbol{kg}} \right)\boldsymbol{=}\frac{\boldsymbol{C}_{\boldsymbol{0}}\left( \frac{\boldsymbol{mg}}{\boldsymbol{L}} \right)\boldsymbol{*}\boldsymbol{f}\boldsymbol{*}\boldsymbol{V}_{\boldsymbol{0}}\left( \boldsymbol{mL} \right)\boldsymbol{*}\boldsymbol{10}^{\boldsymbol{-}\boldsymbol{3}}}{\boldsymbol{m}\left( \boldsymbol{g} \right)\boldsymbol{*}\boldsymbol{10}^{\boldsymbol{-}\boldsymbol{3}}}\boldsymbol{=}\frac{\boldsymbol{C}_{\boldsymbol{1}}\left( \frac{\boldsymbol{mg}}{\boldsymbol{L}} \right)\boldsymbol{*}\boldsymbol{V}_{\boldsymbol{0}}\left( \boldsymbol{mL} \right)\boldsymbol{*}\boldsymbol{10}^{\boldsymbol{-}\boldsymbol{3}}}{\boldsymbol{m}\left( \boldsymbol{g} \right)\boldsymbol{*}\boldsymbol{10}^{\boldsymbol{-}\boldsymbol{3}}}$ **(1)**

$\boldsymbol{W}\left( \boldsymbol{\%} \right)\boldsymbol{=}\frac{\boldsymbol{C}_{\boldsymbol{x}}\boldsymbol{(}\frac{\boldsymbol{mg}}{\boldsymbol{kg}}\boldsymbol{)}}{\boldsymbol{10}^{\boldsymbol{6}}}\boldsymbol{*}\boldsymbol{100}\boldsymbol{\%}$ **(2)**

m_0_(g)：the mass of the sample taken when analyzing the sample; V_0_(mL)_：_After sample digestion, the volume of the fixation; f: factor of dilution; Co(mg/L)：Testing the concentration of solution elements; C_1_(mg/L, C_1_(mg/L)=C_O_(mg/L)*f)：Elemental concentration of sample digestion solution raw solution; C_x_(mg/kg): Final test results for the elements measured; C(mg/kg): Average of the results of three tests for the element measured; W(%):Final test results for the elements measured.

**Tabel S4.** After stability test, ICP-OES test results of the electrolyte solution

| Elements | C_x_ (mg/L) | C (mg/L) | W % |
| --- | --- | --- | --- |
| Fe | <0.05 | <0.05 | 0.000005 |
|  | <0.05 |  |  |
| Rh | <0.05 | <0.05 | 0.000005 |
|  | <0.05 |  |  |

The test subjects were electrolyte solutions after stability testing of FeO_x_/Rh metallene. The results indicate that Rh and Fe elements showed negligible dissolution in the electrolyte.

**Video S1.** Small-scale overall water splitting device.

[Video S1.mp4](Video%20S1.mp4)

**Video S2.** Small-scale overall water splitting device(The multimeter is clamped at the electrode position).

[Video S2.mp4](Video%20S2.mp4)
